# Genomic configuration of *Bacillus subtilis* (NMB01) unveils its antiviral activity against *Orthotospovirus arachinecrosis* infecting tomato

**DOI:** 10.3389/fpls.2025.1517157

**Published:** 2025-03-04

**Authors:** M. Gayathri, R. Sharanya, P. Renukadevi, Varagur Ganesan Malathi, Amalendu Ghosh, Saranya Nallusamy, S. Varanavasiappan, S. Nakkeeran, Saad Alkahtani

**Affiliations:** ^1^ Department of Plant Pathology, Centre for Plant Protection Studies, Tamil Nadu Agricultural University, Coimbatore, India; ^2^ Retired, Coimbatore, India; ^3^ Insect Vector Laboratory, Advanced Centre for Plant Virology, Indian Agricultural Research Institute, New Delhi, India; ^4^ Department of Plant Molecular Biology and Bioinformatics, Centre for Plant molecular Biology and Biotechnology, Tamil Nadu Agricultural University, Coimbatore, India; ^5^ Department of Plant Biotechnology, Centre for Plant Molecular Biology and Biotechnology, Tamil Nadu Agricultural University, Coimbatore, India; ^6^ Department of Plant Pathology, Agriculture College and Research Institute, Kudumiyanmalai, Pudukottai, India; ^7^ Department of Zoology, College of Science, King Saud University, Riyadh, Saudi Arabia

**Keywords:** GBNV, qPCR, whole genome, pan-genome, OrthoVenn

## Abstract

*Orthotospovirus arachinecrosis* (groundnut bud necrosis virus, GBNV) infecting tomato is a devastating viral pathogen responsible for severe yield losses of up to 100%. Considering the significance of the plant growth-promoting bacteria to induce innate immunity, attempts were made to evaluate the antiviral efficacy of *Bacillus subtilis* NMB01 against GBNV in cowpea and tomato. Foliar application of *B. subtilis* NMB01 at 1.5% onto the leaves of cowpea and tomato followed by challenge inoculation with GBNV significantly reduced the incidence of GBNV from 80% to 90% in response to the untreated inoculated control. Hence, we had a quest to understand if any genes were contributing toward the suppression of GBNV in assay hosts. To unveil the secrecy, whole-genome sequencing of *B. subtilis* NMB01 was carried out. The genome sequence of NMB01 revealed the presence of secondary metabolite biosynthetic gene clusters, including non-ribosomal peptide synthetases (NRPSs) and polyketide synthases (PKSs) which also encoded bacteriocins and antimicrobial peptides. The pan-genome analysis identified 1,640 core genes, 4,885 dispensable genes, and 60 unique genes, including MAMP genes that induce host immune responses. Comparative genome and proteome analysis with other genomes of *B. subtilis* strains in a public domain through OrthoVenn analysis revealed the presence of 4,241 proteins, 3,695 clusters, and 655 singletons in our study isolate. Furthermore, the NMB01-treated tomato plants increased the levels of defense-related genes (MAPKK1, WRKY33, PR1, PAL, and NPR1), enhancing immune system priming against GBNV infection. These findings suggest that *B. subtilis* NMB01 can be used as a promising biological control agent for managing plant viral disease sustainably.

## 1 Introduction

Tomato (*Solanum lycopersicum*) is one of the most important cultivated crops of the Solanaceae family, highly valued for its nutritional benefits and economic significance (Frusciante et al., 2007). However, the cultivation of this vital crop is perennially threatened by a myriad of pathogens, among which viral infections significantly hamper tomato production and productivity (Hanssen et al., 2010; Kasmi et al., 2020). Groundnut bud necrosis virus (GBNV), belonging to the genus *Orthotospovirus* in the family *Bunyaviridae* (Satyanarayana et al., 1996), is a devastating virus that causes bud blight disease in tomato and causes a yield loss of up to 100% (Kunkalikar et al., 2011; Mandal et al., 2012). Early infection resulted in chlorotic and necrotic lesions on the leaves and drying of young buds followed by stem necrosis and stunting. Furthermore, necrotic ring spots appear on unriped fruits, and chlorotic ringspots were noticed in ripened fruits (Raja and Jain, 2006). The challenges involved in managing GBNV are compounded by the inefficacy and unsustainability of conventional management strategies. Chemical pesticides targeting thrips, the primary vector of GBNV, are not effective in curbing the virus spread and also pose environmental hazards and contribute to the development of pesticide-resistant thrips populations. This predicament has catalyzed a shift toward exploring biological control strategies, particularly in the utilization of beneficial microorganisms like *Bacillus* species, which offer a more sustainable and environmentally benign approach to disease management (Karadeniz et al., 2006; Gamalero and Glick, 2011). *Bacillus* species are known for plant growth-promoting properties and also have demonstrated significant potential to mitigate plant viral diseases (Damayanti and Katerina, 2008; Vinodkumar et al., 2018). Induction of systemic resistance (ISR) is one of the primary mechanisms through which bacteria confer protection in plants, wherein *Bacillus* spp. trigger a heightened state of defensive readiness throughout the plant (Vinodkumar et al., 2018; Vanthana et al., 2022; Gayathri et al., 2024). This induced resistance is broad spectrum in nature, providing enhanced protection against a variety of pathogens, including viruses. For instance, *B. subtilis* synthesize signaling molecules that activate the plant’s defense pathways, thereby fortifying the banana bunchy top virus infections in banana (Kavino et al., 2007). In addition to ISR, *Bacillus* species serve as a repository of secondary metabolites with potent antiviral activities. They are lipopeptides and antibiotics that have the ability to inhibit viral replication and dissemination within plant tissues (Choudhary and Johri, 2009). Furthermore, foliar application of *B. subtilis* reduces the severity of viral diseases in tomato, a testament to its dual role in inducing systemic resistance and producing antiviral compounds (Wang et al., 2018). Based on these, the current study harnesses the potential of *B. subtilis* NMB01 in combating GBNV, and it is also imperative to delve into their genetic underpinnings, for which the complete genome sequencing of *B. subtilis* NMB01 was carried out to unveil an array of genes, pathways, and metabolites involved in the antiviral action against GBNV. The genetic diversity and adaptability of *B. subtilis* can be comprehensively understood through pan-genome analysis, to identify core, accessory, and unique genes distributed across the strains. This approach, augmented by OrthoVenn analysis, enabled a comparison of the proteome between various *B. subtilis* strains. By identifying the genetic pathways and molecular mechanisms that confer antiviral properties, we can develop targeted biocontrol strategies to mitigate GBNV. These strategies will not only curb the impact of GBNV on tomato but also enhance the sustainability and resilience of agricultural practices. Ultimately, leveraging the natural capabilities of *Bacillus* species presents a promising frontier in the quest to secure global tomato production against the scourge of viral pathogens. Considering the significance of *Bacillus* spp. in the mitigation of GBNV, the present investigation was executed to explore the antiviral nature of NMB01 for inducing the immune response in tomato against GBNV.

## 2 Materials and methods

### 2.1 Strain isolation

The bacterial isolates *Bacillus haynesii* IBHB1, *B. amyloliquefaciens* IBHB2, *Stenotrophomonas maltophila* IBHB3, *B. subtilis* IBHB4, *Pseudomonas aeruginosa* IBHB5, and *B. subtilis* NMB01 were obtained from the Department of Plant Pathology, Tamil Nadu Agricultural University, Coimbatore. The NCBI accession numbers based on 16S ribosomal DNA are given in **Table 1**. Pure cultures of the bacteria were maintained in sterile Petri plates containing Luria–Bertani medium by the single streak method. The plates were incubated at a room temperature of 28°C ± 2°C.

**Table 1:** Testing the antiviral efficacy of bacterial isolates against GBNV in cowpea upon simultaneous inoculation.

Treatment	16S ribosomal DNA accession ID	Mean number of lesions/leaf^a^	Percent inhibition over control
*Bacillus haynesii* IBHB1	PP805691	2.86 (9.73)	82.73
*Bacillus amyloliquefaciens* IBHB2	PP805857	3.86 (11.32)	76.69
*Stenotrophomonas maltophila* IBHB3	PP805902	2.00 (8.12)	87.92
*Bacillus subtilis* IBHB4	PP805943	1.10 (6.01)	89.73
*Pseudomonas aeruginosa* IBHB5	PP806173	2.50 (9.09)	84.90
*Bacilllus subtilis* NMB01	NZ_JALHRZ000000000*	0.86 (5.32)	94.81
Untreated control		16.56 (24.02)	
CD (5%)		0.30	
SE (d)		0.14	

Values in parentheses are arcsine-transformed values.

^a^Mean value represents three replications and each replication contains five plants.

*Bacillus subtilis NMB01 genome accession (BioProject PRJNA817627).

### 2.2 Assessment of antiviral efficacy by bacterial antagonists

#### 2.2.1 Source of virus inoculum

The DeTo GBNV isolate (OR159681) from our previous study (Gayathri et al., 2024) was maintained in a local lesion host, cowpea (VBN3), through sap inoculation for further study. The virus was confirmed as GBNV using N gene-specific primers (GBNV N-F 5′ATGTCTAACGT(C/T) AAGCA(A/G)CTC3′ and GBNV-N-R 5′TTACAATTCCAGCGAAGGACC3′) (Vanthana et al., 2019) through RT-PCR. GBNV-infected tomato plants were used as positive control. As a negative control, healthy cowpea samples were used.

#### 2.2.2 Antiviral nature of the bacterial isolates against GBNV in cowpea

Cowpea seedlings (VBN3) were used to screen the antiviral nature of bacterial isolates against GBNV. Throughout the study, screening was done using the viral isolate DeTo (OR159681) from the Devarayapuram location of Coimbatore, Tamil Nadu Province. As per our previous study, simultaneous inoculation with 1.5% cell suspension culture of bacterial isolates performed well in reducing the disease incidence of GBNV in tomato. Furthermore, the concentration of GBNV was consistently maintained at 1.40 OD (A405 nm) as per the protocol described by Hull (2009) and Vinodkumar et al. (2018). Simultaneous (co-inoculation of the virus and bacterial isolates) inoculations were carried out at a concentration of 1.5% (volume by volume) with cell suspension culture of bacteria at 10^8^ cfu/mL followed by inoculation of GBNV. The plants were kept in insect-proof cages and maintained in glasshouse conditions. The expression of symptoms was observed regularly. Reduction of disease caused by GBNV and inhibition over control were computed. The experiment was performed with three replications and five plants per replication. The best bacterial agent was selected based on their efficiency in reducing the number of local lesions.

#### 2.2.3 Quantification of virus titer

Direct antigen coating enzyme-linked immunosorbent assay (DAC-ELISA) was employed to quantify the viral titer in the bacterized cowpea plants as per the standard procedure of Hobbs et al. (1987) using polyclonal antisera of GBNV obtained from ICRISAT, Hyderabad, India. Samples were collected 4 days after post-inoculation of GBNV. The secondary antibody, anti-rabbit IgG (Cat. No. 1100180011730, Sigma, Germany), was used at a dilution of 1:5,000, and the primary antibody was used at a dilution of 1:10,000. The readings were recorded after an incubation time of 30 min. For each treatment, there were three biological replications and two technical replications. The virus samples (GBNV) maintained in the glasshouse served as a positive control, and healthy cowpea samples served as a negative control. Samples were considered to be positive, according to Clark and Adams (1977), if their absorbance at 405 nm was twice that of the healthy control.

#### 2.2.4 Testing the antiviral efficacy of NMB01 against GBNV in tomato

During an initial screening in cowpea, the bacterial isolate NMB01 demonstrated the highest antiviral activity among the isolates tested. Consequently, NMB01 was tested for its ability to combat GBNV in tomato plants (hybrid Saaho) by simultaneous inoculation. The bacterial isolate NMB01 was sprayed at 1.5% concentration (volume by volume) of crude culture and then GBNV was inoculated as per the standard procedure (Hull, 2009; Vinodkumar et al., 2018). The virus concentration was maintained uniformly at 1.40 OD (A405 nm). The experiment was performed with five replications and five plants per replication. The plants were incubated in an insect-proof chamber, and periodic observations were made for the expression of symptoms in assay host plants, the number of days taken for symptoms expression, and the severity of the disease, following the protocol outlined by Vanthana et al. (2019). Additionally, to evaluate the growth-promoting activity of the NMB01, the entire root system was cut into 30-cm sections. Each section was carefully arranged in a transparent tray (20 × 15 × 2 cm; length × width × height) containing water to minimize root overlap and scanned using an Epson photo scanner (Epson Perfection V800 with 100 dpi resolution). The scanned root images were analyzed with the WinRHIZO Pro image system (Regent Instruments, Inc. - Quebec City, Canada) to measure the average root diameter (RD), total root length (RL), total root surface area (RS), number of crossings, number of tips, number of forks, project area, and total root volume (RV). DAC-ELISA was performed to compare the virus titer between different bacterized plants challenged with GBNV, untreated virus inoculated, and uninoculated plants in tomato. In each treatment, individual plants were tagged for each replication, and leaf samples were collected from the same plants at three time intervals of 0, 5, and 10 days post-inoculation (DPI). The experiment included three representative leaf samples per treatment, with two technical replications, along with samples from healthy control.

Furthermore, the absolute quantification of the virus was done through real-time PCR at different days post-inoculation (3, 5, 7, and 9 DPI) of NMB01 and GBNV. Quantification of the virus was assessed by using GBNV nucleocapsid primer (GBNV F - 5′GGACCAGATGACTGGACCTTC, GBNV R - 5′TCGAAGCTG CAGGGACATT3′) (Vanthana et al., 2022) to amplify 167 bp through real-time PCR in the Bio-Rad CFX96 manager. Samples were taken at different time intervals upon simultaneous inoculation of the bioagent (NMB01) and GBNV inoculum through sap inoculation. A total volume of 10 µL reaction contains 5 µL of SYBR Green master mix (KAPA SYBR @FAST for Light Cycler 480, Cat. No. A1250), 10 pmol/µL concentration of forward primer and reverse primer, 2 µL of nuclease-free water, and 1 µL of template cDNA with an amplification cycle of 95°C for 10 min (initial denaturation) and 40 cycles of 95°C for 30 s, 60°C for 30 s, and 72°C for 30 s, followed by standard melting temperature analysis. The viral copy number was quantified by the absolute quantification method using recombinant plasmid DNA containing the GBNV-CP gene in the pGEMT vector. The copy number was calculated by using the formula


$$\begin{array}{c} \text{Y molecules}=\text{X g}/µ\text{l DNA}\times 6.022\\ \times 10^{23}/\left(\text{Base pair of recombinant plasmid}\times 660\right) \end{array}$$

### 2.3 Comprehensive genome assembly and genome analysis

Using the Quick-DNA Fungal/Bacterial kit (D6005) from Zymo Research, the genomic DNA of NMB01 was extracted, and whole-genome sequencing of the extracted DNA was performed by ONEOMICS Pvt Ltd. (https://oneomics.in/) using high-throughput Illumina sequencing technology. The quality of the raw sequencing reads was assessed using FastQC (version 0.11.9) (Andrews, 2010), which analyzed parameters such as sequence quality scores, GC content, per-base sequence quality, adapter content, and overrepresented sequences. The reads were then processed using Trimmomatic v 0.39 (Bolger et al., 2014) with default settings for quality trimming, and adapter sequences corresponding to P1 and other contaminants were removed. Trimmomatic, when used with default settings, applies a series of predefined trimming and quality control steps to sequencing reads. Adapter sequences were removed using the ILLUMINACLIP function, which detects and removes adapters specified in the TruSeq3-PE adapter files. This process allows up to two mismatches in the adapter seed sequence, with a palindrome clip threshold of 30 and a simple clip threshold of 10. Furthermore, the sliding window trimming removes bases with an average quality score below 15 within a sliding window of 4 bases. Low-quality bases at the beginning and end of reads were trimmed using Leading and Trailing trimming, both set to remove bases with quality scores below 3. Reads shorter than 36 bases after trimming were discarded based on the minimum read length setting. These default parameters ensured effective quality control while retaining the integrity of high-quality reads for downstream analysis. Genome assembly was carried out using SPAdes (version 3.11.1), a *de-novo* assembly algorithm tailored for bacterial genomes (Bankevich et al., 2012) with default parameters, and the quality-controlled reads were mapped to the assembled contigs using BWA-MEM v 0.7 17 to verify consistency and coverage. Post-assembly quality checks included QUAST 4.6.1 (Gurevich et al., 2013) to evaluate metrics like N50, number of contigs, and misassemblies and CheckM 1.0.9 (Parks et al., 2015) to assess genome completeness and contamination, ensuring the reliability of the assembled genome.

### 2.4 Annotation of the genome assembly

The assembly of *B. subtilis* NMB01 was submitted to the Comprehensive Genome Analysis Service provided by the Pathosystems Resource Integration Center (PATRIC) (Brettin et al., 2015). The annotation was carried out using the RASTtk pipeline (RASTtk) unique genome identifier of 1452.102 (Brettin et al., 2015) to categorize gene functions based on subsystems. The annotation process identified protein-coding genes, RNA genes, and functional categories, providing a detailed initial annotation of the genome. For a more comprehensive analysis, the annotated genome was further processed using PATRIC (Davis et al., 2020). The PATRIC platform facilitated comparative analysis with related microbial strains, including ortholog detection, phylogenetic reconstruction, and identification of unique biological processes and structural complexes through subsystem annotations. The genome annotation services in PATRIC used the k-mer-based antimicrobial resistance (AMR) gene detection method, which utilizes PATRIC’s curated collection of representative AMR gene sequence variants and assigns to each AMR gene functional annotation, a broad mechanism of antibiotic resistance. Virulence gene detection was performed by screening the genome against the VFDB, PATRIC_VF, and Victors DataBase using the Abricate tool and PATRIC’s integrated tools, with a minimum threshold of 90% coverage and identity (Chen et al., 2005). For secondary metabolite discovery, the genome was analyzed using AntiSMASH 5.1.0 (Blin et al., 2019) with relaxed strictness to identify putative biosynthetic gene clusters (BGCs). Functional annotation of these clusters was carried out through BLASTp (Altschul et al., 1997) and Pfam (Mistry et al., 2021) analysis to determine domain functions and similarities to known clusters. Furthermore, the metabolic pathways associated with the genome were mapped using the Kyoto Encyclopedia of Genes and Genomes (KEGG) database (Kanehisa et al., 2017). This combined approach utilizing RASTtk and PATRIC ensured high-quality genome annotation, comparative genomic insights, and identification of potential virulence factors and secondary metabolites.

### 2.5 Comparative genome analysis of *Bacillus subtilis* NMB01 with other biocontrol strains of *Bacillus subtilis*

To investigate the relationship between *B. subtilis* NMB01 and other *B. subtilis* strains, a comparative genome analysis was conducted. For multi-genome analysis, the whole-genome sequences (WGS) of 19 *B. subtilis* strains*—*BYS2, ZD01, YB-04, XF1, UD1022, TR21, SG6, RS10, GUCC4, MC4, PMB102, BS16, BS16045, BSn5, GQJK2, HD15, J5, JCL16, and KC141*—*have been retrieved from the NCBI database. These strains were selected based on previous reports of their antimicrobial activity, underscoring their significance for this study. The specifics of their antimicrobial properties are provided in **Supplementary Table S1**. Details of genome size, number of genes, and proteins of the above sequences were compared. Multiple genome alignment was performed by constructing Blast Ring Image Generator (BRIG analysis) (Alikhan et al., 2011) and MAUVE 2.4.0 (Darling et al., 2011), to compare the genome of NMB01 with the strains of *B. subtilis*. The open reading frame of NMB01 was compared with the other strains of *B. subtilis*.

### 2.6 Pan-genome analysis

The pan-genome analysis of *B. subtilis* NMB01 was carried out with 19 related strains of *B. subtilis* obtained from the NCBI database. The pan-genome analysis was performed utilizing the PGAP web server (Chen et al., 2018) to identify core genes (present in all strains), accessory genes (present in some strains), and unique genes (specific to NMB01). Clustering of all nucleic acid and protein sequences was analyzed via the GeneFamily (GF) method within the pan-genome analysis pipeline (PGAP). In the GF method, all protein sequences were pooled, followed by all-vs-all sequence alignment using BLASTALL (score: 40; e-value: 1e−10; coverage and identity: 0.5), and clustered using the Markov cluster algorithm (MCL) (Enright et al., 2002; Van Dongen, 2008). Pan-genome profiles were calculated based on orthologous clustering using Heap’s law or an exponential model (Tettelin et al., 2005; Rasko et al., 2008). Additionally, a phylogenetic tree was generated utilizing the pan-based neighbor-joining method and UPGMA algorithms in PHYLIP (Felsenstein, 1989), and the tree was represented using the Interactive Tree of Life (iTOL) v6.5.3 (https://itol.embl.de/). Based on the phylogenetic analysis, the closely related species of *B. subtilis* NMB01 included *B. subtilis* strains YB-04, PMB102, BS16045, HD15, RS10, and GUCC4. A proteome-wide comparison and annotation of clusters of orthologous groups (COGs) were conducted using the OrthoVenn web server (Wang et al., 2015). The analysis was performed using the FASTA sequences of all selected species, with the default e-value cutoff of 1e−5 and an inflation value (−*I*) of 1.5 to identify orthologous clusters.

### 2.7 Effect of flagellin genes on the activation of MAMP-triggered immunity

Based on genome comparison, the number of flagellin genes was higher in NMB01. To examine the effect of flagellin in microbe-associated molecular pattern (MAMP)-triggered immunity, a qPCR study was conducted through real-time PCR in the Bio-Rad CFX96 manager. Samples were drawn at different time intervals (0, 3, 5, 7, and 9 days post-inoculation) following simultaneous inoculation of NMB01 and GBNV in tomato plants. A total volume of 10 µL reaction mixture contains 5 µL of SYBR Green master mix (KAPA SYBR @ FAST for Light Cycler 480, Cat. No. A1250), 10 pmol/µL concentration of forward and reverse primers for the N gene of GBNV, 2 µL of nuclease-free water, and 1 µL of template cDNA with an amplification cycle of 95°C for 10 min (initial denaturation) and 40 cycles of 95°C for 30 s, 60°C for 30 s, and 72°C for 30 s, followed by melting curve analysis. In the present study, actin was used as an internal control since the expression of actin remained stable during virus interaction. The major defense genes (*MAPKK*1, WRKY33B, *PR1*, *PAL*, and *NPR1*) involved in defense activation were selected to study the effect of NMB01 against GBNV. For each defense gene expression study, three biological replications and two technical replications were maintained. The primers used in this study are listed in **Supplementary Table S2**.

### 2.8 Statistical analysis

The fold changes in gene expression were calculated using the formula


ΔΔCt=Δ Ct sample–Δ Ct reference

The relative fold changes in the transcript level were represented graphically by converting the ΔΔCT value to 2^−(ΔΔCt)^ (Livak and Schmittgen, 2001). The statistical analysis for the relative fold change was performed using the TIBCO Spotfire analyst version 7.11.1 280 software.

## 3 Results

### 3.1 Maintenance of the virus inoculum

The virus isolate DeTo (OR158681) was maintained in the local lesion host cowpea (VBN3) through sap inoculation. Initially, chlorotic lesions were observed 4 days after inoculation and later turned into necrotic lesions at 6 DPI (**Supplementary Figure S1**). RT-PCR was carried out from the inoculated samples and the expected amplicon of 830 bp was obtained for the N gene primers of GBNV (**Supplementary Figure S2**).

### 3.2 Testing the antiviral efficacy of bacteria against GBNV in cowpea

Among the bacteria tested, *B. subtilis* NMB01 was effective in reducing the lesion number to 0.86 per leaf which was 94.81% inhibition over the untreated plants. It was followed by *B. subtilis* IBHB4, in which the lesion number was 1.10 per leaf, which was 89.73% reduction over the inoculated control. The lowest inhibition of 76.69% was observed in *B. amyloliquefaciens* (IBHB2)-treated plants. On the other hand, the untreated inoculated plants expressed the maximum number of 16.56 lesions. Furthermore, the virus titer in bacterized (NMB01) cowpea plants was assessed through DAC-ELISA. The results revealed that the inoculated untreated plants had the highest OD value of 1.37 at A405 nm, whereas the NMB01-treated plants had a very low OD value of 0.62. Thus, the results confirmed that the application of NMB01 has reduced the GBNV titer in the local lesion host cowpea (**Table 1**; **Supplementary Figure S3**).

### 3.3 Testing the antiviral efficacy of NMB01 against GBNV in tomato

**Figure 1:**
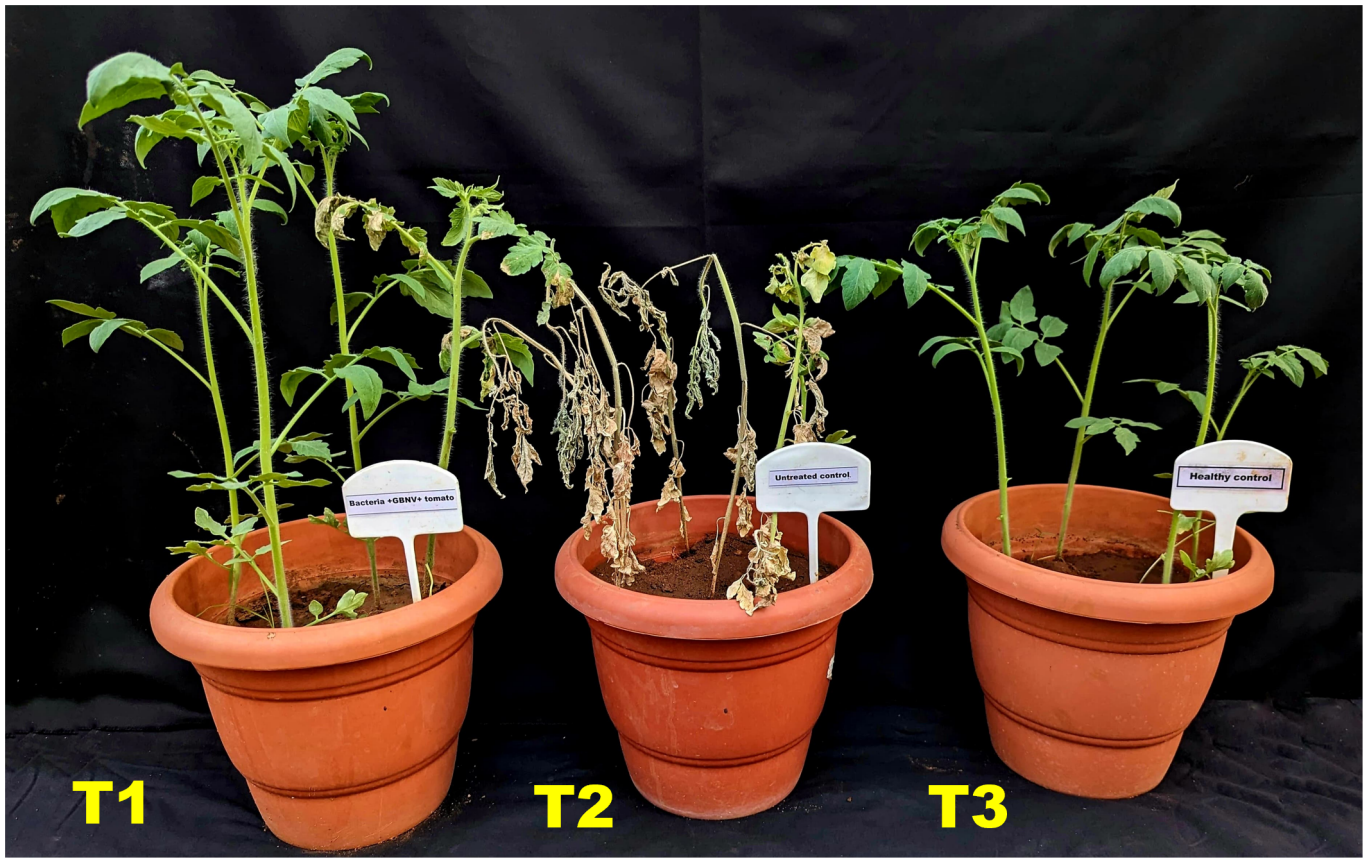
Impact of NMB01 on GBNV infection in tomato. T1- Mild necrotic at 12 DPI; T2- Complete drying of the plant at 12 DPI; T3- No symptom. T1—NMB01-treated; T2—untreated inoculated control; T3—healthy control.

Based on the preliminary screening in cowpea, the isolate NMB01 was selected and screened against GBNV in tomato upon simultaneous inoculation. The number of plants exhibiting the symptom was higher in the untreated plants with 80.00% disease incidence and had a disease severity of 3.12 with chlorotic and necrotic lesions in the leaves and stems compared to bacteria NMB01-treated plants. However, the incidence in NMB01-treated plants was 16% with a disease severity of 0.46. The delay in symptom expression was observed in NMB01-treated plants compared to the untreated plants. The symptom expression in the untreated plants appeared at 7 DPI and the complete drying of the whole plant was observed at 12 DPI. In the case of bacteria-treated plants, the symptom expression was observed only at 10 DPI and the plants were alive with mild necrotic rings (**Figure 1**; **Table 2**).

**Table 2:** Assessment of antiviral efficacy of *Bacillus subtilis* against the infection of GBNV in tomato.

Treatment	Number of symptomatic plants/total number of plants inoculated						% Disease incidence (DPI)	Severity index
	R_1_	R_2_	R_3_	R_4_	R_5_	Total infected plants		
T1—*B. subtilis* NMB01	1/5	0/5	1/5	1/5	1/5	4	16	0.46
T2—untreated inoculated control	3/5	4/5	5/5	4/5	4/5	20	80	3.12
T3—healthy control	0/5	0/5	0/5	0/5	0/5	0	–	–

### 3.4 Plant root growth parameters

**Figure 2:**
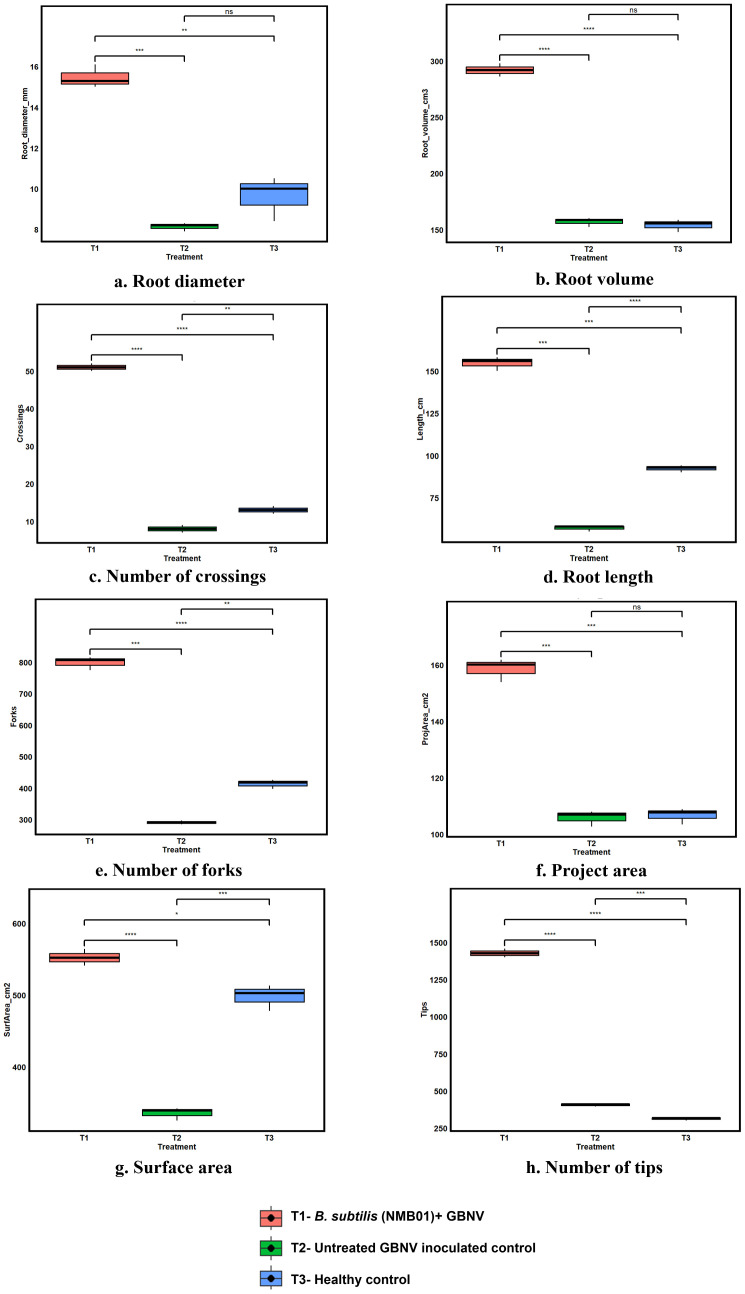
**(a–h)** Root Architecture profiling of NMB01 treated virus-inoculated, untreated and healthy tomato plants Asterisks indicate significance difference between the treatments according to the *t* - test followed by p adjusted value using Bonferroni methods (* for *p* < 0.05,** for *p* < 0.01, *** for *p* < 0.001, **** for *p* = 0.000, ns for non significance).

The application of *B. subtilis* NMB01 has influenced the root growth characteristics of tomato. Compared with the untreated plants, the NMB01 treatment increased root length, surface area, root volume, and root diameter by 67.01%, 51.05%, 54.19%, and 89.61%, respectively. Furthermore, in the case of the untreated virus-inoculated plants, the reduction in root length, surface area, root volume, and root diameter was observed to an extent of 20%–38%. The number of forks (1,429), tips (799), and crossing (51) was also higher in NMB01-treated plants than the untreated plants with an average number of 313 forks, 414 tips, and 13 crossings (**Figures 2a–h**, **Supplementary Figure S4**).

### 3.5 Assessment of GBNV titer by DAC-ELISA in NMB01-treated tomato plants

**Figure 3:**
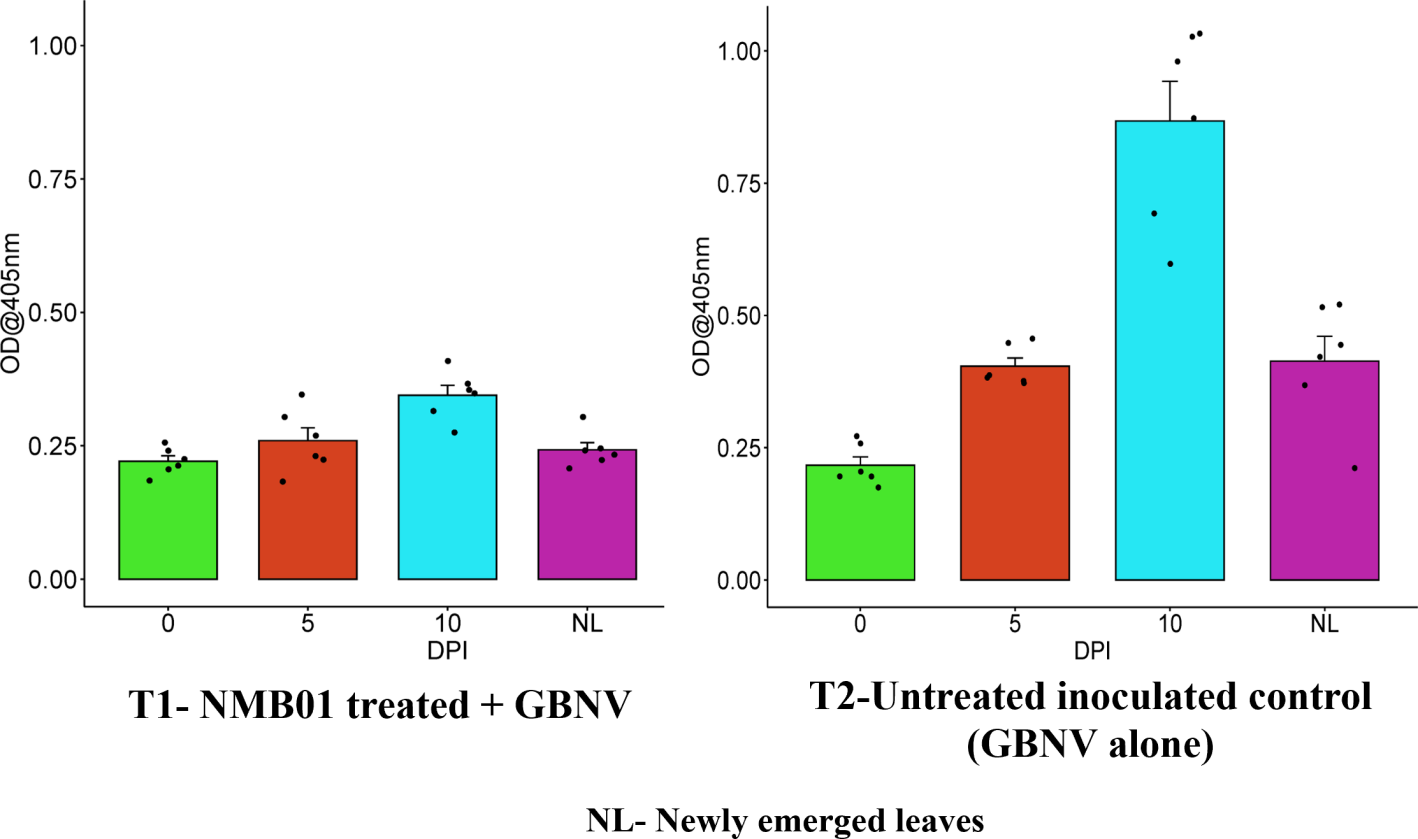
Assessment of GBNV titer in bacteria-treated tomato (Saaho) through DAC-ELISA. Treatment 1—NMB01-treated inoculated; treatment 2—untreated inoculated control; NL—newly emerged leaves.

Tomato leaves bacterized with NMB01 had the lowest OD values of 0.221, 0.259, and 0.344 at 0, 5, and 10 DPI, respectively, whereas in the untreated inoculated control, the OD values were 0.216, 0.404, and 0.867, respectively. In the case of newly emerged leaves, the OD value was the lowest in the treated plants (0.242) compared to the untreated inoculated (0.413) control. The OD value was 0.199 in the healthy control. The virus titer in bacteria-treated plants was not increased until 10 DPI, which confirmed the antiviral nature of NMB01 (**Figure 3**).

### 3.6 Absolute quantification of the virus

**Figure 4:**
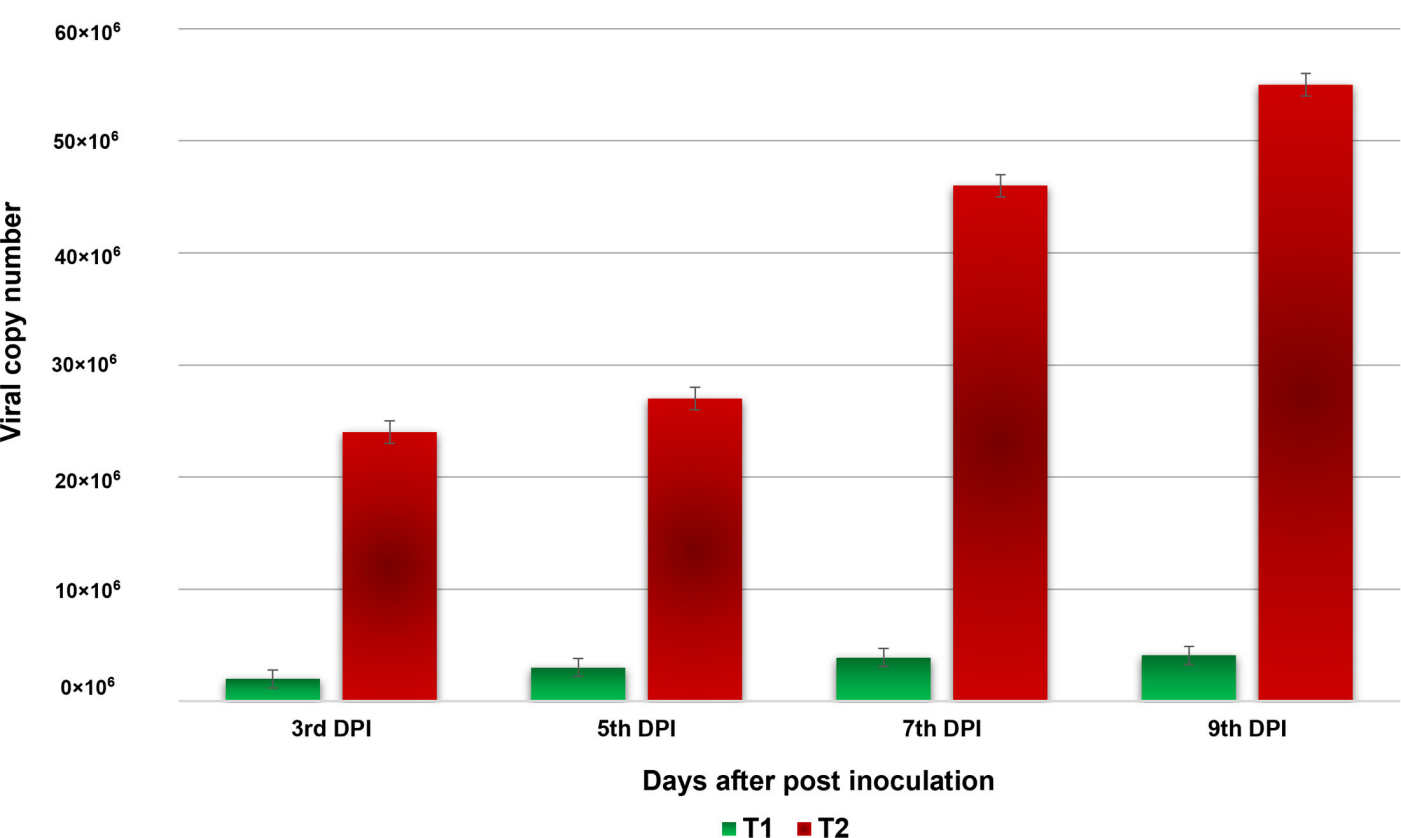
Absolute quantification of GBNV titer in NMB01-treated tomato (Sahoo) through qPCR. Treatment 1—NMB01-treated inoculated; treatment 2—untreated inoculated control.

The virus copy number was measured on different days after virus inoculation on tomato plants (3, 5, 7, and 9 DPI). From 3 to 9 days post-inoculation, the virus load consistently increased in both the bacteria NMB01-treated and untreated tomato plants challenged with GBNV. However, a minimal increase in the virus load was observed in the NMB01-treated plants compared to the untreated inoculated control from 5 DPI onward. In the untreated inoculated control, the viral copies continued to increase from 0 DPI and recorded the highest copy number of 55 × 10^6^, whereas in NMB01-treated plants, the copy number also increased from 0 to 9 DPI but the level was significantly lower (4.1 × 10^6^ at 9 DPI) than the untreated inoculated plants (**Figure 4**).

### 3.7 Genome assembly

Furthermore, whole-genome sequencing was carried out for NMB01 to identify the key gene’s role in the antiviral mechanism. The assembled genome was submitted to the Comprehensive Genome Analysis Service where the genome was annotated using the RAST tool kit (RASTtk). Accordingly, the taxonomy of the genome pertained to Cellular organisms > Bacteria > Terrabacteria group > Firmicutes > Bacilli > Bacillales > Bacillaceae > *Bacillus* > *Bacillus subtilis*. The genome was submitted to the NCBI database and the accession number was NZ_JALHRZ000000000. This genome had 28 contigs, with a total length of 3,985,883 bp and an average G+C content of 43.62%, 4,241 protein-coding sequences (CDS), 69 transfer RNA (tRNA) genes, and 3 ribosomal RNA (rRNA) genes. The assembly details are given in **Table 3A**.

**Table 3A:** Assembly details of NMB01.

Assembly details of NMB01	
Contigs	28
GC content	43.62
Plasmids	0
Contig L50	2
Genome length	3,985,883 bp
Contig N50	982,545
CDS	4241
tRNA	69
rRNA	3

**Table 3B:** Protein features of NMB01.

Protein features	
Hypothetical proteins	672
Proteins with functional assignments	3,569
Proteins with EC number assignments	1,047
Proteins with GO assignments	871
Proteins with pathway assignments	773
Proteins with PATRIC genus-specific family (PLfam) assignments	3,991
Proteins with PATRIC cross-genus family (PGfam) assignments	4,082

During gene prediction, RASTtk annotates 672 hypothetical proteins and 3,569 proteins with functional assignments. The proteins with functional assignments included 1,047 proteins with enzyme commission (EC) numbers, 871 with gene ontology (GO) assignments, and 773 proteins that were mapped to KEGG pathways. PATRIC annotation included two types of protein families, and the genome had 3,991 proteins that belong to the genus-specific protein families (PLFams) and 4,082 proteins that belong to cross-genus protein families (PGFams) (**Table 3B**). The annotated genome of NMB01 is displayed as a circular graphical representation explaining GC content, GC skew, presence of contigs, CDS, RNA genes, virulence genes, and coding sequences displaying the antimicrobial genes (**Supplementary Figure S5**).

### 3.8 Subsystem analysis

Analysis of the subset was carried out through the PATRIC annotation to reveal the presence of superclasses with varying numbers of subsystems (SS) and associated families/genes. The result revealed that the majority of the genes accounted for metabolism processes followed by cellular processes and energy (**Supplementary Figure S6A**). Other subsystems such as protein processing and stress response, defense, and virulence also contributed significantly to the gene count. Subsystems like regulation and cell signaling, miscellaneous, and cell envelope had the fewest genes. Metabolism encompassed 98 subsystems with a total of 761 genes. Protein processing included 43 subsystems and 225 genes, while stress response, defense, and virulence comprised 33 subsystems with 128 genes. Cellular processes consisted of 29 subsystems containing 254 genes, and energy involved 25 subsystems with 214 genes. DNA processing had 17 subsystems and 88 genes. Membrane transport included 16 subsystems with 77 genes, and RNA processing covered 13 subsystems with 52 genes. The cell envelope category had 5 subsystems and 27 genes. Both miscellaneous and regulation and cell signaling categories had 4 and 3 subsystems, respectively, each with 11 genes (**Supplementary Figure S6B**). Using Blast2GO annotation (Conesa et al., 2005) in OmicsBox 2.0., a functional categorization by gene ontology (GO) terms was conducted. The analysis was based on the Blastx hits from the non-redundant database. In total, 17 GO terms related to biological processes, 5 GO terms related to cellular components, and 9 GO terms related to molecular function classes were identified.

### 3.9 Gene coding for secondary metabolites

Out of 28 contigs, 10 contig regions were coded for secondary metabolites, namely, contigs 1, 2, 3, 4, 6, 7, 8, 13, 14, and 15. The number of regions predicted to encode secondary metabolites varied between the contigs. In contig 1, six regions were identified that coded for secondary metabolites including bacillibactin, subtilin, subtilosin, and thailanstatin with a similarity of 100% to the reference genus *B. subtilis* subsp*. subtilis*. For contig 2, three regions coded plipstatin and each one for contigs 3, 4, 6, 7, 8, 13, 14, and 15. For the other predicted metabolites, fengycin was detected in contig 8, surfactin in contigs 7 and 15, and plipstatin in contigs 14 and 15. The nucleotide range and the percent similarity are presented in **Supplementary Table S3**. Furthermore, the genes present in the genome underwent several pathways, mainly for metabolisms, cellular processes, and environmental signaling. More than 180 pathways were identified for metabolisms and approximately 30–60 for other processes (**Supplementary Figure S7A**). Genes involved in secondary metabolite underwent various metabolic pathways, notably terpenoid backbone biosynthesis pathway, fatty acid metabolism, aminoacyl-tRNA biosynthesis pathway, ubiquinone and other terpenoid-quinone biosynthesis, biotin metabolism, phenylalanine metabolism, glycolysis/gluconeogenesis, nitrogen metabolism, pyruvate metabolism, peptidoglycan biosynthesis, amino sugar and nucleotide sugar metabolism, starch and sucrose metabolism, glyoxylate and dicarboxylate metabolism, and riboflavin metabolism. The total pathways are represented in **Supplementary Figure S7B**.

### 3.10 Antimicrobial resistance genes

*Bacillus subtilis* NMB01 possesses a diverse repertoire of genes involved in various AMR mechanisms, which potentially contributed to its antimicrobial activity. The majority of these shared genes were associated with different mechanisms of antimicrobial resistance. It included genes related to antibiotic inactivation enzymes, proteins involved in altering cell wall charge, antibiotic target protection and replacement protein and efflux pump conferring antibiotic resistance, and regulators that modulate the expression of antibiotic resistance genes (**Table 4**). The genes were also responsible for the survival of bacteria under extreme environmental conditions.

**Table 4:** Antimicrobial resistance gene in NMB01.

AMR mechanism	Genes
Antibiotic inactivation enzyme	ANT(6)-I, FosB, Vgb(B)
Antibiotic target in susceptible species	Alr, Ddl, dxr, EF-G, EF-Tu, folA, Dfr, folP, gyrA, gyrB, inhA, fabI, Iso-tRNA, kasA, MurA, rho, rpoB, rpoC, S10p, S12p
Antibiotic target modifying enzyme	RlmA(II)
Antibiotic target protection protein	BcrC
Antibiotic target replacement protein	fabL
Efflux pump conferring antibiotic resistance	BceA, BceB, EbrA, EbrB, Lmr(B), Tet(L), YkkCD
Gene conferring resistance via absence	GidB
Protein altering cell wall charge conferring antibiotic resistance	GdpD, MprF, PgsA
Regulator modulating expression of antibiotic resistance genes	BceR, BceS, LiaF, LiaR, LiaS

### 3.11 Comparative genome analysis

The genomic attributes [genome size, gene count, coding DNA sequences (CDSs), protein-coding genes, and G+C content] of *B. subtilis* NMB01 were analyzed and compared to 19 other *B. subtilis* strains available in the NCBI database. The total gene count across these strains ranged from 3,921 to 4,437. Among them, strain GUCC4 exhibited the highest number of total genes (4,437), of which 4,250 were coding genes. In contrast, the strain from our study, *B. subtilis* NMB01, possesses a total of 4,220 genes, with 4,044 CDSs (**Table 5**).

**Table 5:** Comparative genome analysis of 20 *Bacillus subtilis* isolates.

Genome code	Accession	Genome size	Genes (total)	CDSs (total)	Genes (coding)	CDSs (with protein)	CDSs (without protein)	Genes (RNA)	tRNAs	ncRNAs	Pseudogenes
BSn5	NC_014976.1	4,093,599	4,241	4,122	4,211	4,211	101	119	53	5	101
HD15	CP080508	4,173,431	4,426	4,305	4,208	4,208	97	121	86	5	97
R31	TP046591	4,186,872	4,343	4,222	4,145	4,145	77	121	86	5	77
YB-04	CP072525	4,156,177	4,276	4,154	4,085	4,085	69	122	87	5	69
TR21	CP046592	4,105,857	4,229	4,108	4,058	4,058	50	121	86	5	50
NMB01	NZ-JALHRZ000000000	3,985,883	4,220	4,143	4,044	4,044	99	77	69	5	99
SG6	CP009796	4,079,669	4,233	4,123	4,022	4,022	101	110	84	5	101
JCL16	CP054177.1	4,101,682	4,179	4,058	3,984	3,984	74	121	86	5	74
GQJK2	CP020367	4,072,961	4,190	4,069	3,976	3,976	93	121	86	5	93
PMB102	CP047645	4,103,088	4,231	4,110	3,964	3,964	146	121	86	5	146
ZD01	CP046448	4,015,360	4,101	3,995	3,929	3,929	66	106	80	5	66
XF-1	NC_020244	4,061,186	4,185	4,077	3,922	3,922	155	108	76	5	155
BYS2	CP074571	4,030,791	4,118	3,997	3,917	3,914	83	121	86	5	83
J5	CP018295	4,117,900	4,130	4,011	3,917	3,917	94	119	87	5	94
UD1022	NZ_CP011534	4,025,326	4,133	4,012	3,900	3,900	112	121	86	5	112
KC14-1	CP103328	3,908,079	4,140	4,019	3,895	3,895	124	121	86	5	124
BS-916	CP009611	3,981,674	3,921	3,741	3,675	3,675	66	121	86	1	66
BS16045	CP017112	4,165,121	4,355	4,234	4,076	4,076	158	121	86	5	158
GUCC4	CP139440	4,215,739	4,437	4,316	4,250	4,250	66	121	86	5	66
MC4-2	CP147492	4,076,630	4,221	4,100	3,975	3,975	125	121	86	5	125

**Figure 5:**
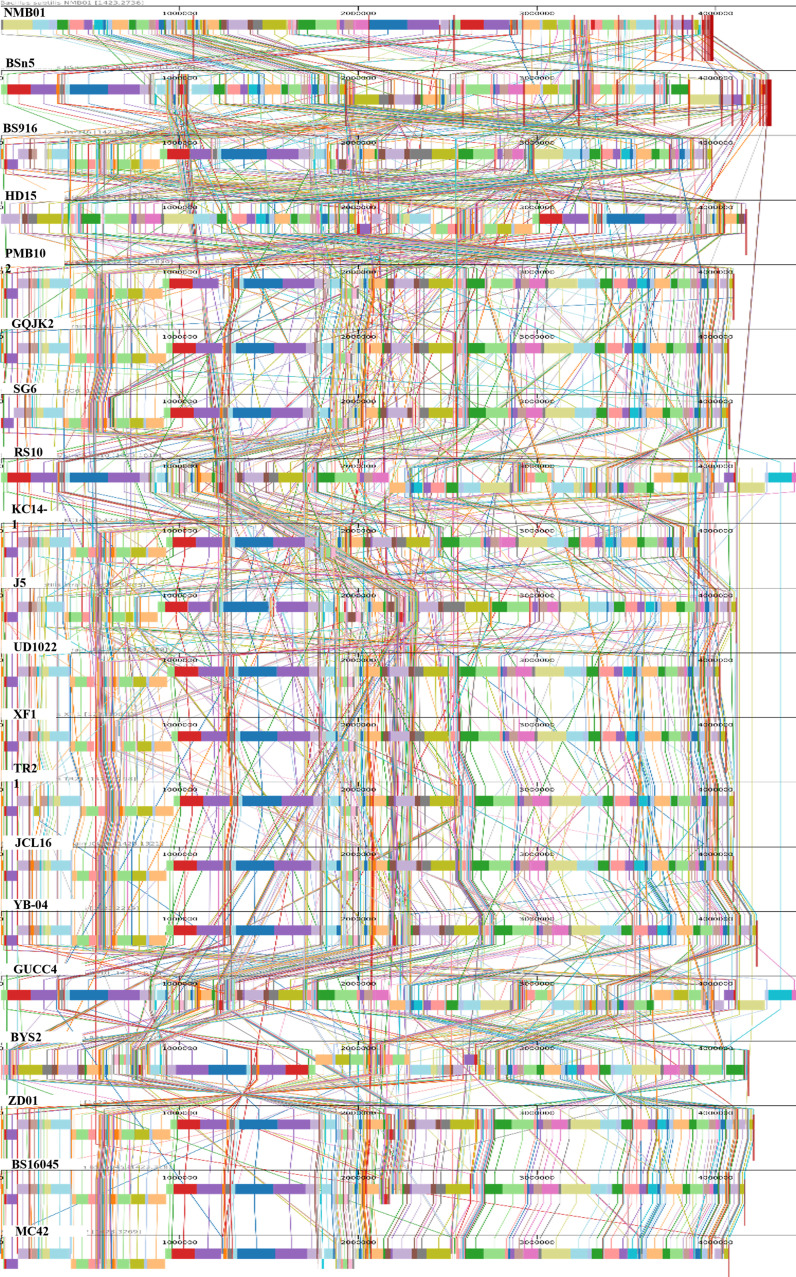
Comparison of NMB01 genome sequences with other 19 *Bacillus subtilis* using MAUVE. By using MAUVE, pairwise alignments of genomes were generated. Each genome is laid out horizontally with homologous segments outlined as colored rectangles. Rearrangements are shown with colored lines. Boxes with the same color indicate a locally collinear block (LCB) or homologous region (syntenic regions) shared among genomes. The scale is given in nucleotides.

To further explore the genomic characteristics of NMB01, a genome synteny analysis was performed. Through the analysis, aligned multiple genomes demonstrated a substantial degree of synteny between NMB01 in comparison with the other species. Using the MAUVE alignment tool, a synteny map was produced, highlighting local collinear blocks (LCBs) and various translocation regions. Each LCB signified instances of horizontal gene transfer among the genetically similar species. Homologous regions, represented as locally collinear blocks (LCBs) across the genomes, indicated that NMB01 shared extensive synteny with the majority of the analyzed strains. The consistent alignment of these LCBs suggested a high level of conservation, reinforcing the idea that NMB01 possessed a core genomic structure common to antimicrobial-producing *B. subtilis* strains (**Figure 5**).

**Figure 6:**
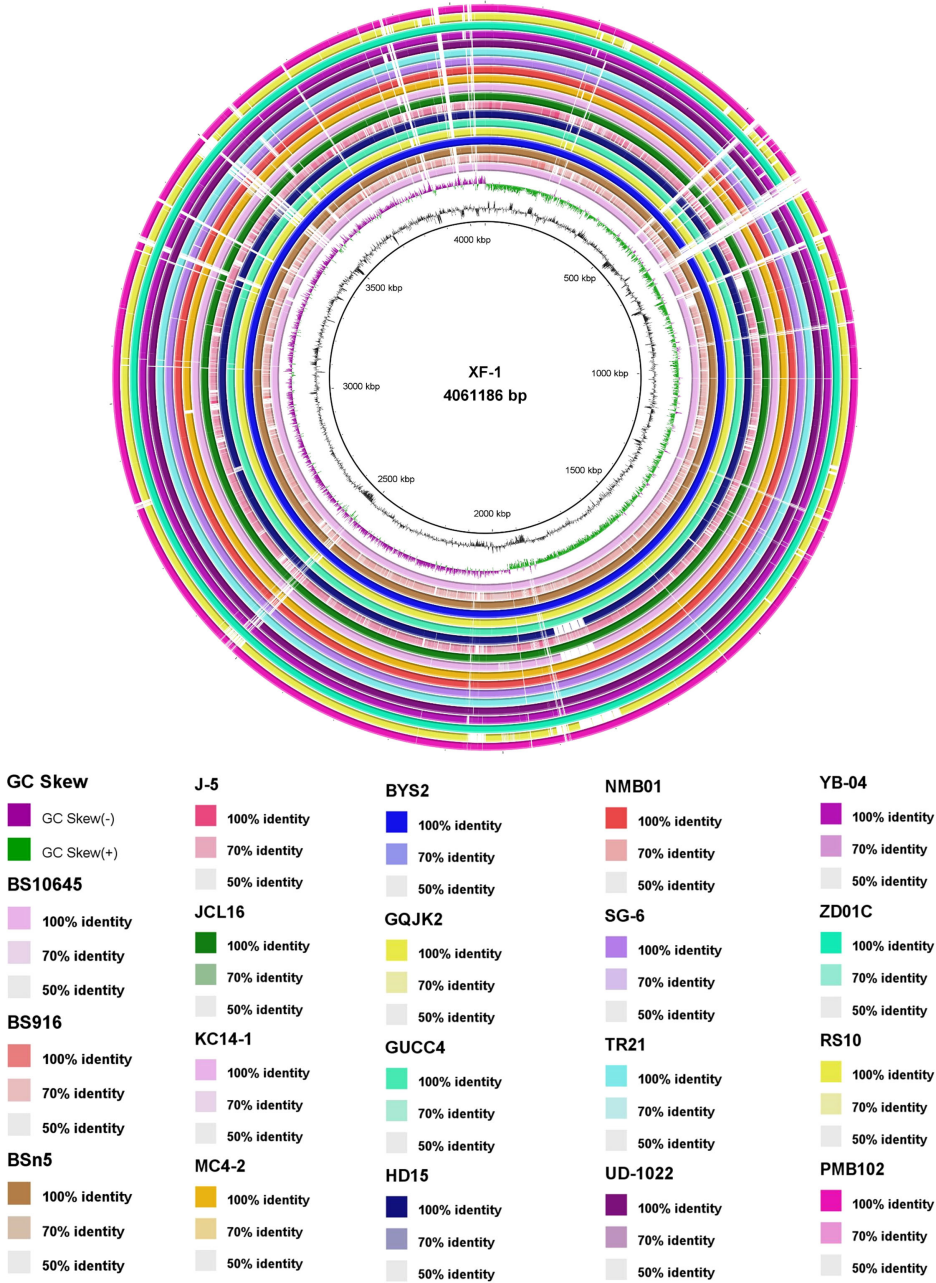
Blast Ring Image Generator (BRIG) output image of 20 *B subtilis* genome. The genome was aligned using BRIG, which displays percent G+C, GC skew, and homology based on BLASTn (from inner to outer). Color saturation indicates the homology rate, and blanks (white regions) show the absence of similarity. *Bacillus subtilis* XF1 is used as a reference strain.

The comparative genomic analysis of *B. subtilis* strains using BRIG provided an in-depth visualization of conserved and divergent regions among multiple related strains, with homology levels ranging from 50% to 100%. Notably, the circular genome comparison highlighted that strain NMB01 exhibited a high degree of similarity with the other 18 strains, which are known for their antimicrobial activity (**Figure 6**). This high similarity suggests that NMB01 shares a conserved genomic backbone with these strains, potentially encoding similar sets of genes involved in antimicrobial compound biosynthesis, regulatory pathways, and environmental adaptation.

### 3.12 Pan-genome analysis

**Figure 7:**
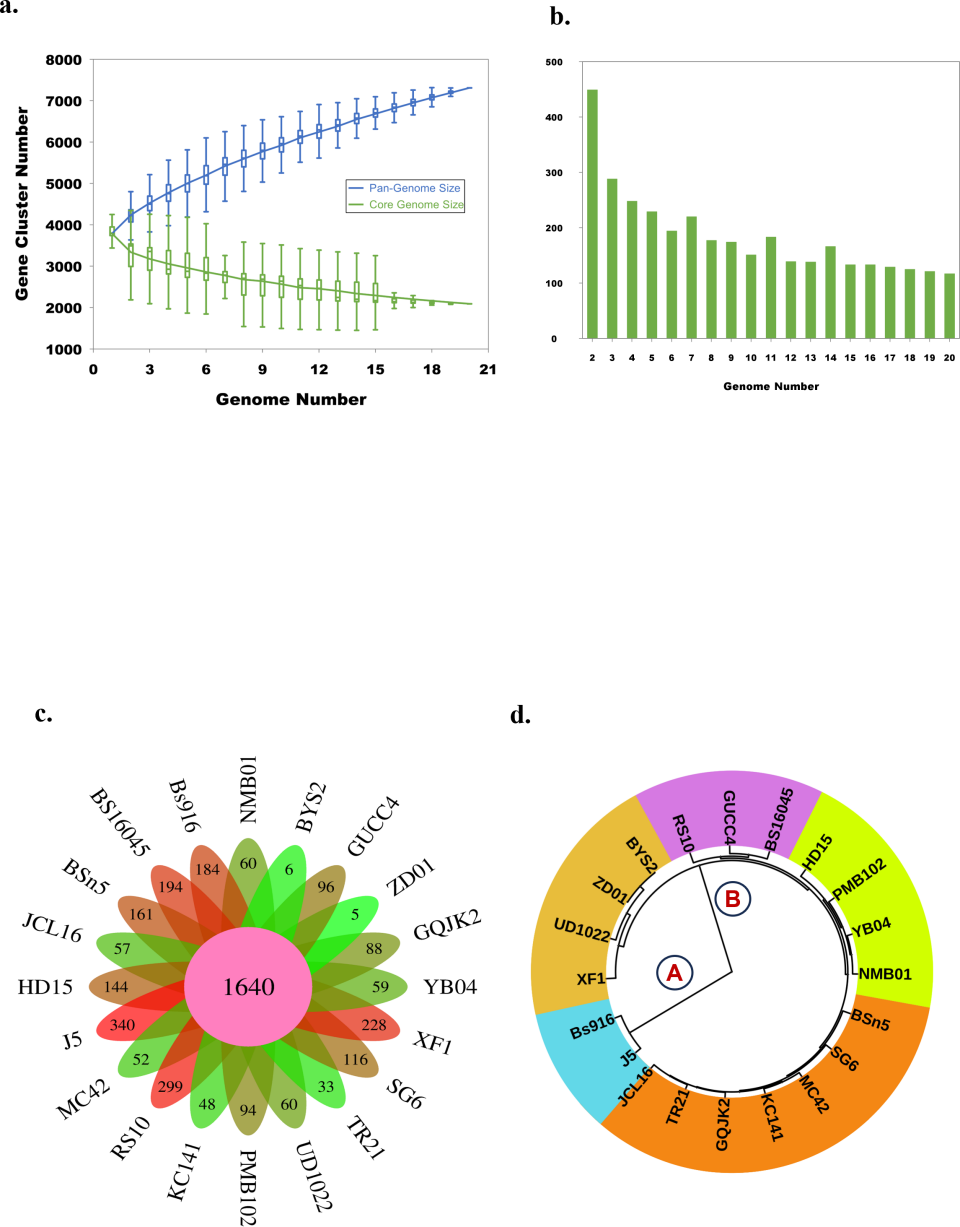
Pan-genome analysis of *Bacillus subtilis* in PGAP. **(a)** Gene accumulation curves of the pan-genome (blue) and core-genome (green) of 20 *B subtilis* genome. The blue boxes denote each genome’s *B subtilis* pan-genome size for comparison. The green boxes show the *B subtilis* core genome size for each genome for comparison. **(b)** Graph showing the number of new gene clusters with an increase in the number of *B subtilis* genomes. **(c)** Flower plot illustrating the number of core genes (in the center) and strain-specific genes (in the petals) found in each genome. **(d)** Phylogenetic tree of *B subtilis* using the pan-based neighbor-joining method.

**Figure 8:**
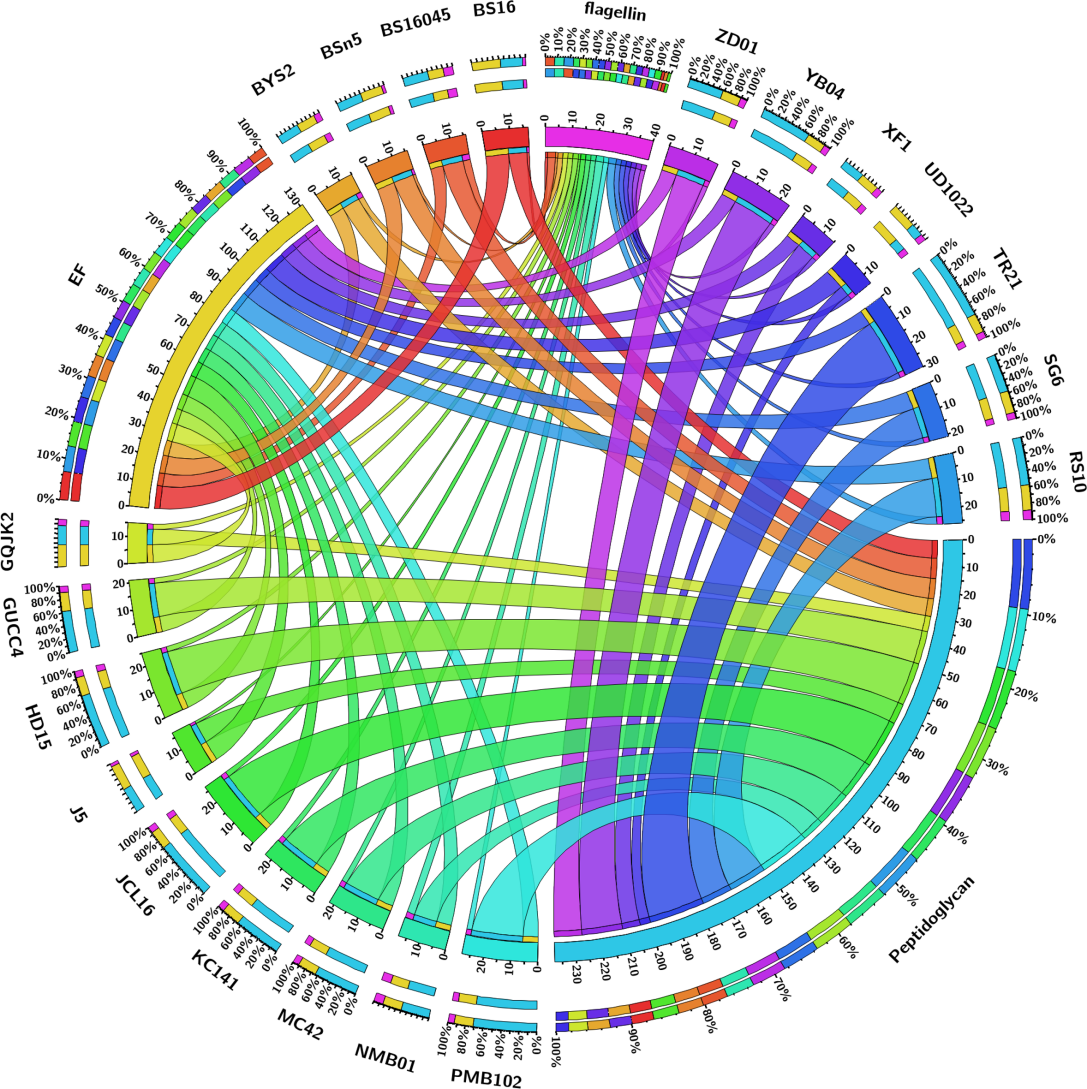
A Circos plot depicting the various MAMP genes (peptidoglycan and elongation factor).

**Figure 9:**
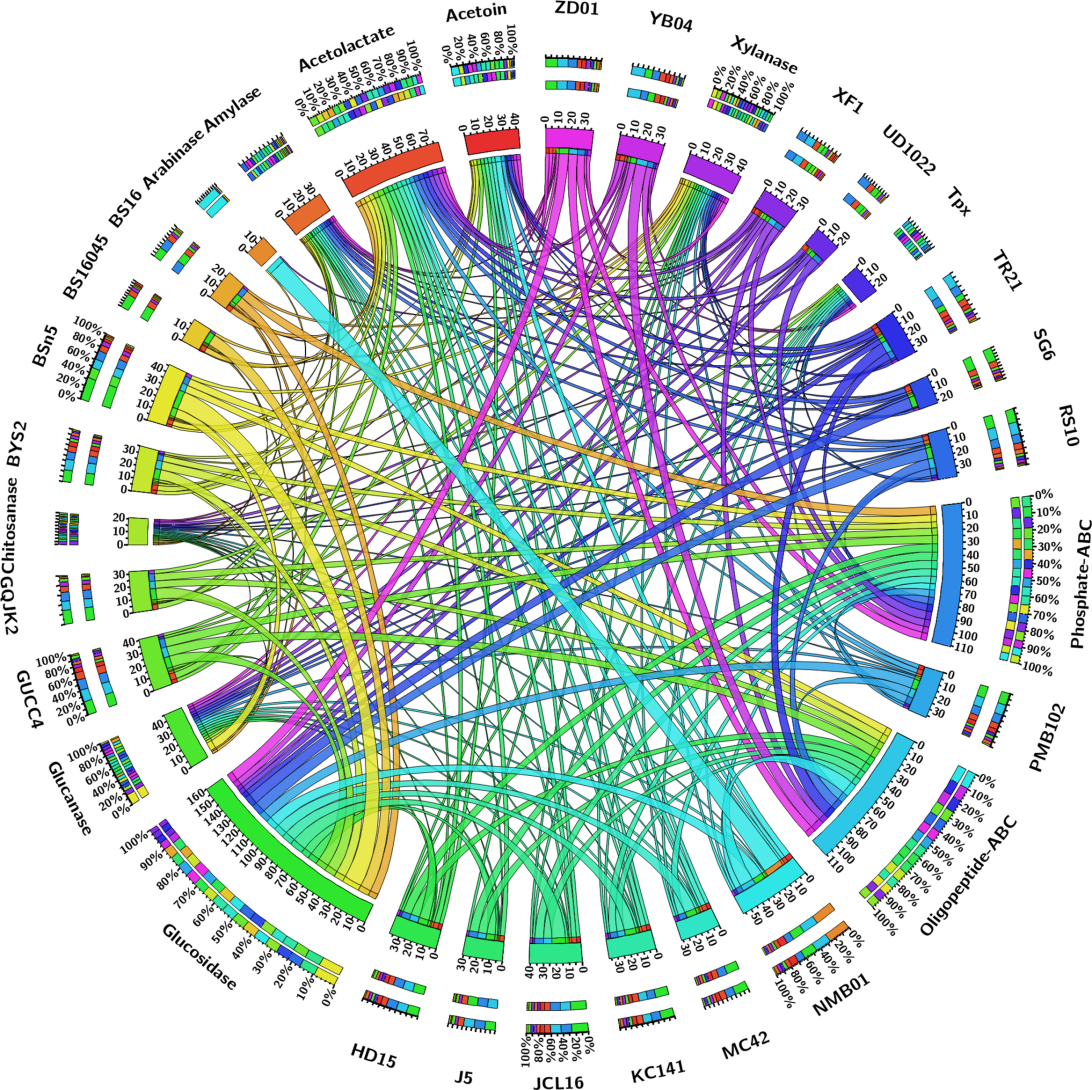
A Circos plot depicting the various hydrolytic genes (amylase, arabinose, xylanase, thiol peroxidase, glucosidase, glucanase, chitinase, chitin-binding protein, and ABC transporter).

The pan-genome analysis of *B. subtilis* NMB01, along with 19 other strains, depicted the pan-genome as a blue curve and the core genome as a green curve. When the number of gene families was plotted against increasing genome number, a rise in the pan-genome size and a decrease in core-genome size were observed. This implicated that the pan-genome for the studied isolate was an “open form” concept of Heap’s law (Tettelin et al., 2005), indicating that the number of gene families expanded with the addition of *B. subtilis* genomes, suggesting that *B. subtilis* can easily acquire new genes (**Figures 7a, b**). The comparison of core orthologous genes across the strains identified 1,640 core genes (19%) and 4,885 dispensable genes (55%). The unique genes accounting for 26% across the 20 genomes ranged from 38 in *B. subtilis* ZD01 to 340 in *B. subtilis* J5 (**Supplementary Figure S8**). Using a pan-genome-based neighbor-joining method, the 20 *B. subtilis* were categorized into two clusters, labeled A and B. Among these, strains BS916 and J5 formed a distinct cluster. Strain NMB01 was placed in cluster B, closely related to the YB-04, HD15, and PMB102 strains (**Figures 7c, d**). Furthermore, the genes associated with MAMP, glucanase, amylase, xylanase, glucosidase, thiol peroxidase, and chitin-binding proteins were compared. In our strain NMB01, we identified the presence of the following genes: 2 glucanase genes, 2 xylanase genes, 2 amylase genes, 9 glucosidase genes, 2 thiol peroxidase genes, and 15 ABC transporter genes. The highest number of arabinose genes (*n* = 15) found in our study isolate was followed by BSn5 and XF1 (*n* = 1), while it was absent in the rest of the species compared. Notably, our isolate also possessed the highest number of MAMP genes (*n* = 15). Of these, six were associated with elongation factors, nine with peptidoglycan, and three with flagellin. Interestingly, the flagellin gene count in isolate NMB01, as well as in isolates RS10 and BS16045, was higher than the other compared isolates (**Figures 8**, **9**; **Supplementary Tables S4**, **S5**).

### 3.13 OrthoVenn analysis

**Figure 10:**
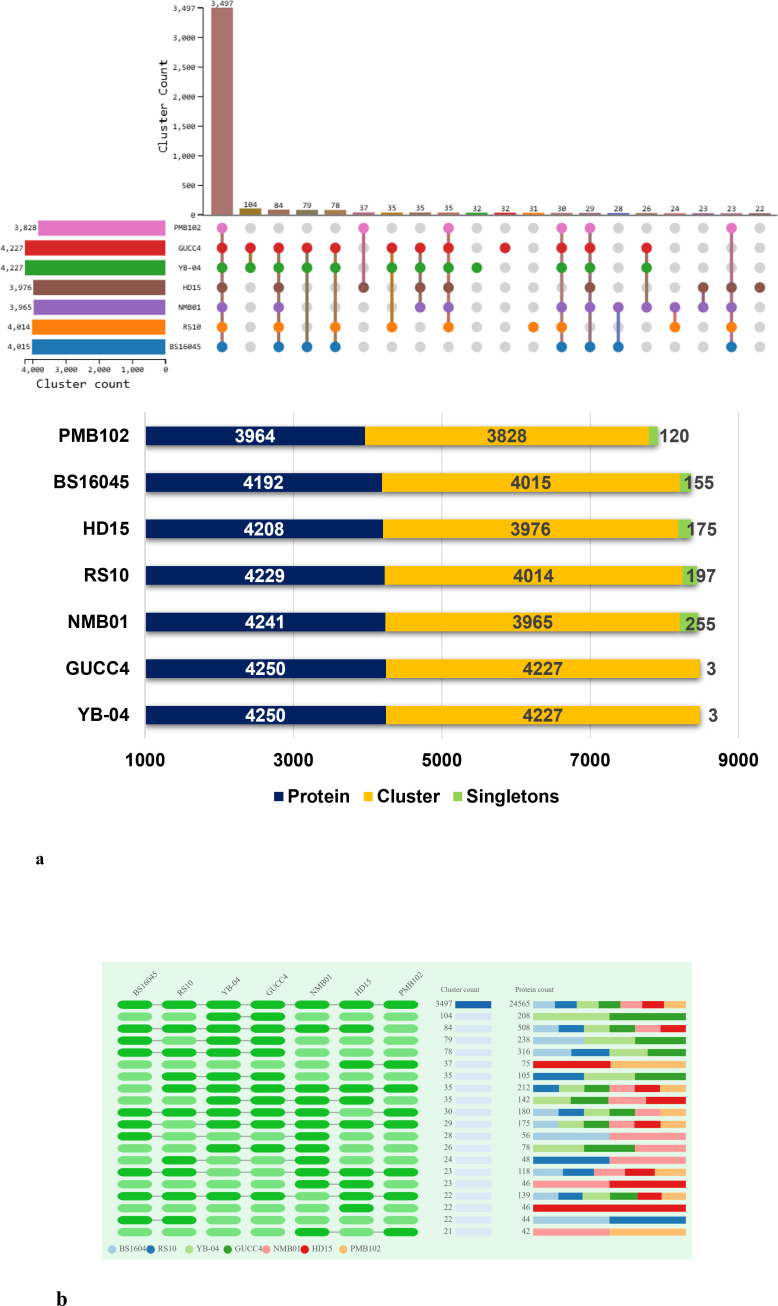
Orthologous cluster analysis of *Bacillus subtilis* spp. **(a)** An UpSet plot displaying unique and shared orthologous clusters among the species. The left horizontal bar chart shows the number of orthologous clusters per species, while the right vertical bar chart shows the number of orthologous clusters shared among the species. The lines represent intersecting sets. **(b)** Overlays identified by the OrthoVenn3 analysis of *B subtilis* species.

Orthologous cluster analysis revealed the presence of 4,547 clusters of orthologous proteins. These clusters contained proteins from 20 *B. subtilis* species, and the proteins within each cluster were related by a common ancestral gene. Out of the 4,547 clusters, 3,482 are single-copy clusters, i.e., each cluster containing one protein from each species. There were 908 singletons representing 3.10% of all clusters. The 59 overlaps suggested the shared orthologous proteins across these clusters, indicating potential functional similarities or common evolutionary origins. In comparison to other strains, NMB01 contained 4,241 proteins, 3,965 clusters, and 255 singletons (**Figures 10a, b**; **Table 6**).

**Table 6:** Proteome-wide comparison of *Bacillus subtilis* NMB01 and related strains based on orthologous cluster analysis.

Species	Proteins	Clusters	Singletons
YB-04	4,250	4,227	3
GUCC4	4,250	4,227	3
NMB01	4,241	3,965	255
RS10	4,229	4,014	197
HD15	4,208	3,976	175
BS16045	4,192	4,015	155
PMB102	3,964	3,828	120

### 3.14 Effect of flagellin genes on the activation of MAMP-triggered immunity in tomato

**Figure 11:**
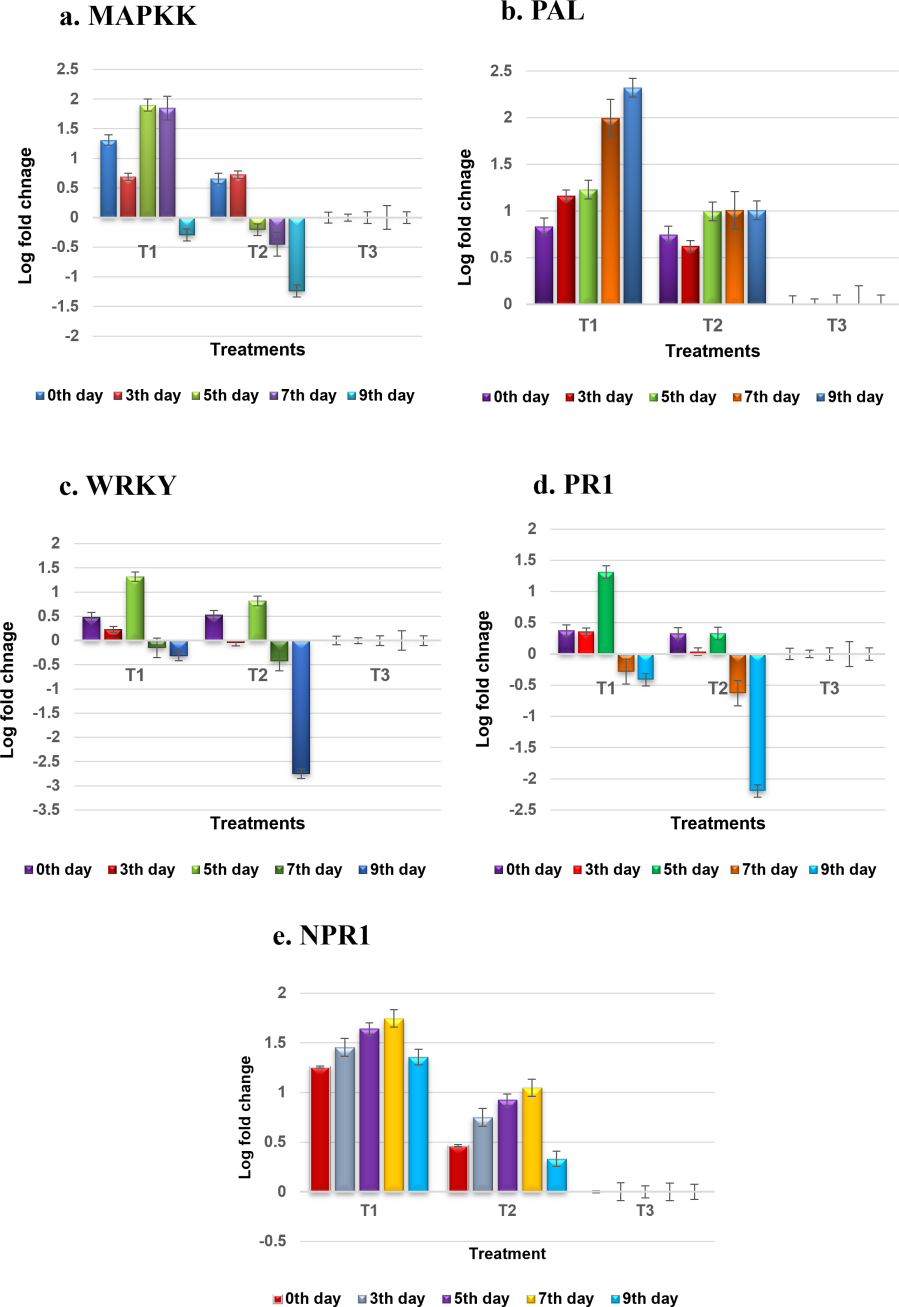
**(a–e)** Expression profiling of defense genes during tritrophic interaction of GBNV, NMB01, and tomato. T1—NMB01 treated; T2—untreated inoculated control; T3—healthy control.

The relative gene expression between bacteria NMB01-treated and untreated control plants of tomato was assessed using the comparative 2^−ΔΔCT^ method. The actin gene was used as a reference gene. Inoculation with GBNV prompted an increase in the expression of the *MAPKK* gene, beginning at 0 DPI. At this initial time point, the relative fold increase in *MAPKK* expression was 1.309-fold compared to mock-inoculated plants. This upregulation continued, with transcript levels rising to a peak of 1.9-fold at 5 DPI, slightly tapering to 1.8-fold at 7 DPI, and then declined at 9 DPI (**Figure 11a**). Conversely, in the untreated plants, the *MAPKK* transcript levels presented a different pattern. At 0 DPI, the expression level was 0.659-fold, which slightly increased to 0.725-fold at 3 DPI. However, this was followed by a sharp decline to −0.2-fold at 5 DPI, further decreasing to −1.239-fold subsequently. These results revealed a stark divergence in the *MAPKK* gene expression in response to GBNV inoculation between the treated and untreated plants. The treated plants disclosed a significant initial upregulation, which was sustained over several days before tapering off. The untreated plants, however, exhibited a slight initial increase in expression, followed by a dramatic downregulation.

The relative fold level of the phenylalanine ammonia lyase (*PAL*) gene in plants treated with bacteria NMB01 showed a consistent increase from the day of inoculation to 10 DPI. Initially, at 0 DPI, the *PAL* gene expression level was at −0.83-fold. This expression increased to 1.16-fold at 3 DPI and further rose to 1.22-fold at 5 DPI. The upward trend continued, reaching a peak at 10 DPI with a maximum expression level of 2.32-fold. Notably, there was no decline in the *PAL* gene expression levels throughout this period. In contrast, the untreated control plants also showed an increase in *PAL* gene expression, but the levels were consistently lower compared to the treated plants. The initial expression level in the untreated plants was 0.74-fold. At 10 DPI, the expression increased to 1.00-fold. However, despite this increase, the expression levels in the untreated plants did not reach the same as those in the treated plants at any observed time point. The *PAL* gene, involved in the phenylpropanoid pathway, also exhibited elevated transcript levels in bacteria-treated plants, suggesting an augmented production of defense-related compounds (**Figure 11b**).

Upon virus inoculation, the *WRKY*33 gene exhibited a dynamic expression pattern in the treated plants. Initially, the expression level was upregulated to 0.48-fold, but this upregulation was transient and declined at 3 DPI. At 5 DPI, the *WRKY*33 expression reached its peak at 1.31-fold but subsequently decreased at 7 and 9 DPI. At 7 and 10 DPI, the expression levels dropped to 0.15-fold and −1.31-fold, respectively. In contrast, the *WRKY* 33 transcript levels in the untreated, virus-infected plants were significantly lower. Immediately following infection, the expression level was 0.53-fold, which then sharply declined to −0.04-fold at 3 DPI. This decline continued and was −2.75-fold at 10 DPI. These observations suggested a differential regulatory mechanism of the *WRKKY* 33 gene in response to virus inoculation, with the treated plants resulting in an initial upregulation followed by fluctuating expression levels. However, the untreated plants displayed a consistent and obvious downregulation of *WRKY*33 over the observed period (**Figure 11c**). Likewise, the *MAPKK* and *WRKY* genes, being the integral components of signal transduction pathways in plant immunity, demonstrated significant transcriptional increases of defense proteins. *MAPKK*s play a vital role in amplifying defense signals, while *WRKY*33 transcription factors are key regulators of defense gene expression.

The transcript level of *PR1* in bacteria NMB01-treated plants increased immediately after virus inoculation to 0.37-fold. This upregulation continued, reached 1.3-fold at 5 DPI. However, at 9 DPI, the expression level declined to −0.41-fold. In contrast, the untreated plants also exhibited an increase in *PR1* expression immediately after virus infection, reaching 0.33-fold. This expression level was maintained up to 5 DPI, but subsequently, it declined sharply at 9 DPI to −2.19-fold. The *PR1* gene, commonly associated with systemic acquired resistance, showed a marked increase in the treated plants, indicating an enhanced defense response (**Figure 11d**).

As anticipated, the level of *NPR1* was upregulated upon GBNV inoculation in the treated plants. *NPR1*, a central regulator of salicylic acid-mediated defense responses, was similarly upregulated, highlighting its role in coordinating the immune response. The transcript level of *NPR1* increased progressively from 1.25-fold at 0 DPI to 1.74-fold at 7 DPI. However, at 9 DPI, this upregulation declined slightly to 1.35-fold. In contrast, plants inoculated with GBNV alone unveiled a downregulation of *NPR1* transcripts over the same period. Starting at 0 DPI, the *NPR1* transcript level was lower at 0.46-fold and continued to decrease, reaching 0.33-fold at 10 DPI. It indicated that the initial response to GBNV inoculation in the treated plants involved an increase in *NPR1* levels and was maintained up to 7 DPI. On the other hand, the *NPR1* levels in plants inoculated with GBNV alone consistently declined, signifying a higher virus infection compared to the treated plants (**Figure 11e**).

Overall, the results revealed that inoculation with GBNV in NMB01-treated plants elicited a significant upregulation in the transcript levels of several key genes, including *PR1*, *PAL*, *NPR1*, *MAPKK*, and *WRKY*. This obvious increase in gene expression is likely attributable to the interaction with the flagellin gene, which in turn activates MAMP-triggered immunity.

## 4 Discussion

Plant viral diseases are like plant cancers and account for half of the plant diseases resulting in a threat to food security and economic instability among the farming community (Anderson et al., 2004). *Orthotospovirus arachinecrosis* (GBNV) in tomato is one of the devastating viral pathogens in tomato that leads to 100% yield loss depending on the stage of infection and severity. The use of chemicals to manage viral diseases in plants often leads to environmental pollution which has made it necessary to search for an alternative management strategy. Biological control using *Bacillus* spp. is considered to be an alternative strategy of plant disease management. In the current study, the antiviral efficacy of *Bacillus* spp. against GBNV on cowpea and tomato was evaluated. The number of lesions in cowpea was significantly reduced in NMB01-treated plants, and the virus titer was also reduced compared to the untreated inoculated plants. In tomato, the plants treated with NMB01 showed reduced disease severity and virus accumulation when compared to inoculated plants without any treatments. Many reports showed that the application of some *Bacillus* spp. increased plant growth and reduced viral infections (Sorokan et al., 2020; Gangireddygari et al., 2023). El-Gendi et al. (2022) reported that the foliar application of *B. subtilis* HA1 culture filtrate enhanced the growth parameter in tomato and reduced the disease severity of *Tobamovirus tabaci* infection. Similarly, various reports endorsed the application of *Bacillus* spp., reducing the viral infection of *Ilarvirus TSV* in cotton (Vinodkumar et al., 2018), *T. tabaci* and *Potyvirus capsivenamaculae* in pepper (Damayanti and Katerina, 2008), *Orthotospovirus tomatomaculae* in tomato (Beris et al., 2018), *Potyvirus phaseovulgaris* in cowpea (Udaya Shankar et al., 2009), *Potexvirus ecspotati* and *Potyvirus yituberosi* in potato (Veselova et al., 2022), and *Potyvirus capsivenae* in pepper (Xuan et al., 2024). The virus titer was reduced and the expression of the symptom was also delayed in the case of NMB01-treated plants. Similarly, Gangireddygari et al. (2023) reported that the foliar application of *P. putida* and *B. licheniformis* reduced the virus titer of pepper mild mottle virus up to 43%–47%. Similarly, Rajaofera et al. (2020) reported that the application of *B. atrophaeus* HAB5 reduced the virus titer of *T. tabaci* in tobacco. Furthermore, the application of the bacteria NMB01 improved the root architecture profiling of tomato. These are in line with the findings of Araujo et al. (2021), who reported that the application of *B. subtilis* and *B. japonicum* changed the growth and root architecture in soybean. *Bacillus subtilis* modified the partitioning of assimilates in soybean with an increase in root biomass and positive changes in root architecture.

Our study further hearsays the whole-genome sequence of NMB01 to determine its taxonomic position and to study the molecular basis of mechanisms involved in the antiviral nature. The antiviral nature of NMB01 can be attributed to several molecular mechanisms identified through genomic analysis. Firstly, the presence of secondary metabolite biosynthetic gene clusters, particularly non-ribosomal peptide synthetases (NRPSs) and polyketide synthases (PKSs), advocates the production of antiviral compounds. These metabolites are known to interfere with viral replication and assembly, providing a broad-spectrum antiviral effect. Moreover, the genome encodes for various bacteriocins and antimicrobial metabolites including surfactin, fengycin, and bacillibactin. The secondary metabolites produced by *B. subtilis* NMB01 are predicted to play significant roles in the observed antiviral activity. These metabolites are known for their diverse bioactivities, including antimicrobial and plant defense-eliciting properties. Genes related to the synthesis of lipopeptides such as surfactin and fengycin have demonstrated antiviral properties against CMV in pepper through the induction of defense response (Kang et al., 2021). Similarly, Hussein et al. (2016) reported the antiviral and stimulator ability of surfactin as a plant defense response by decreasing symptom severity against *Tobamovirus tomatotessellati* in tomato through the activation of two genes PAL and BGL2 involved in salicylic acid (SA) and jasmonic acid (JA) pathways, respectively. In addition, Li et al. (2023) reported that surfactin, fengycin, and bacillibactin produced by *B. amyloliquefaciens* exhibit antimicrobial activity against *P. yituberosi* in potato plants, highlighting their potential role in suppressing viral pathogens. Numerous studies employing the comparative genome analysis of *Bacillus* spp. revealed that the genome harbored multiple gene clusters that are involved in various biological, cellular, and metabolic activities involved in growth promotion and antimicrobial activity (Grady et al., 2019; Xu et al., 2020; Liu et al., 2021; Zaid et al., 2022). Similarly, the research by Saravanan et al. (2021) unveiled that the genome of *B. velezensis* VB7 (CP047587) was characterized with MAMP genes including elongation factor, flagellin, and non-ribosomal peptide synthetase gene clusters responsible for the antifungal activity against *Fusarium oxysporum cubense*. Furthermore, the secondary metabolites of NMB01 were predicted using anti-SMASH and revealed that the six regions in contig 1 code for secondary metabolites including bacillibactin, subtilin, subtilosin, bacilysin, and thailanstatin. Similar to our results, Ma et al. (2018) analyzed the whole genome of *B. atrophaeus* and reported 13 secondary metabolite gene clusters responsible for biological resistance mediated through surfactin, fengycin, pelgipeptin, xenocoumacin, bacillomucin, and rhizocticin. Surfactin and fengycin are well-known for their roles as elicitors of induced systemic resistance (ISR) in plants, as demonstrated in several studies (Romero et al., 2007; Ongena et al., 2010). But whether these genes are conserved in all the genome of different isolates of *B. subtilis* was the next issue raised in our investigation. Hence, to understand the distribution of these genes, a comparative evaluation of the genome of *B. subtilis* was attempted through pan-genome analysis. It was used to explore the diversity of core, pan, and unique genes between the specific species (Vernikos et al., 2015). In the present study, we conducted the pan-genome analysis of NMB01 with other 19 available *B. subtilis* isolates from the NCBI database. It indicated the diversity of core genes, accessory genes, and unique genes. Specifically, our strain NMB01 contains 1,640 core genes, 4,885 dispensable genes, and 60 unique genes. Similar to our study, Kadiri et al. (2023) analyzed the pan-genome of *B. velezensis* VB7 with 53 other *B. velezensis* isolates. They reported that 30 unique genes were present in VB7. These findings revealed the versatility of VB7 in controlling plant pathogens and induced innate immunity in plants against multiple domains of pathogens. From the genome analysis of NMB01 in comparison with the other 19 strains, we identified the presence of MAMP genes, such as flagellin, elongation factor, and peptidoglycan, which are responsible for triggering the immune response in the host. This defense response in plants was mediated by SA or JA pathways (Rajamanickam and Nakkeeran, 2020; Vanthana et al., 2022). Several studies have demonstrated that the application of beneficial bacteria can effectively induce systemic resistance in plants against a variety of pathogens (Choudhary and Johri, 2009; Srinivasan and Mathivanan, 2009; Wang et al., 2009; Pieterse et al., 2014; Joshi et al., 2021; Gayathri et al., 2024; Karthikeyan et al., 2024). Furthermore, the critical role of plant defense enzymes in enhancing disease resistance has also been extensively reported (Prasannath, 2017; Ji et al., 2020; Xu et al., 2021). In the current study, the tomato plants treated with NMB01 exhibited a significant increase in the accumulation of defense-related proteins and genes, including Phenylalanine Ammonia-Lyase (*PAL*), Pathogenesis-Related protein 1 (*PR1*), Non-expressor of Pathogenesis-Related genes 1 (*NPR1*), WRKY transcription factors, and Mitogen-Activated Protein Kinase (*MAPKK*). Similarly, the induction of defense enzyme activities in the leaves following soil inoculation with *B. subtilis* YB-04 and *Fusarium oxysporum* f. sp. *cucumerinum* (Foc) highlights the activation of defense enzymes including polyphenol oxidase (PPO), superoxide dismutase (SOD), catalase (CAT), PAL, and lipoxygenase (LOX), resulting in systemic resistance mechanism (Xu et al., 2022). This suggested that NMB01 effectively primed the tomato plants’ immune system, enhancing their ability to combat GBNV infection through robust activation of their innate defense mechanisms. Thus, our investigation has paved the way for exploring *B*. *subtilis* NMB01 to induce the immune response to quench GBNV infection in tomato.

## 5 Conclusion

This study highlights the potent antiviral properties of *B. subtilis* NMB01 against GBNV in tomato, by significantly reducing disease incidence and virus titer. Whole-genome sequencing of *B. subtilis* NMB01 revealed the presence of biosynthetic gene clusters responsible for producing key antiviral metabolites, including NRPSs and PKSs, and antimicrobial peptides such as bacilysin, surfactin, fengycin, and bacillibactin. Comparative genomic and pan-genome analyses identified critical genes and pathways that promote plant growth and enhance immune responses. The ability of *B. subtilis* NMB01 to suppress viral disease through the activation of bioactive compounds and plant defense mechanisms underscores its potential as a sustainable biocontrol agent. With further validation through large-scale field trials, *B. subtilis* NMB01 could be developed into a cost-effective and eco-friendly solution for managing viral diseases in tomato and other crops.

## Data Availability

The datasets presented in this study can be found in NCBI database and accession numbers can be found in the article. The original contributions presented in the study are publicly available in NCBI. This data can be found here: ["https://www.ncbi.nlm.nih.gov/bioproject/?term=PRJNA817627"Bacillus subtilis strain:NMB01 (ID 817627) - BioProject - NCBI].
