# Supplementary material for: Genomic configuration of Bacillus subtilis (NMB01) unveils its antiviral activity against Orthotospovirus arachinecrosis infecting tomato
Source: Front Plant Sci. 2025 Mar 4;16:1517157. doi: 10.3389/fpls.2025.1517157 (PMC11913681; doi:10.3389/fpls.2025.1517157)
Supplement: Supplementary file 1 [file DataSheet1.pdf]

Supplementary Material

Fig S1. Maintenance of virus inoculum in cowpea (VBN3)

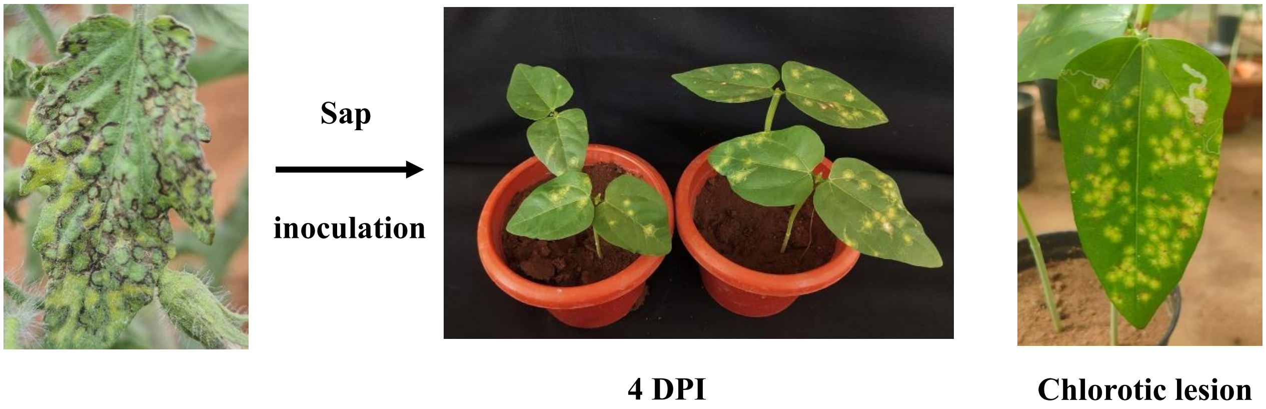

Fig S2. Agarose gel electrophoresis of RT-PCR product of GBNV Nucleocapsid gene

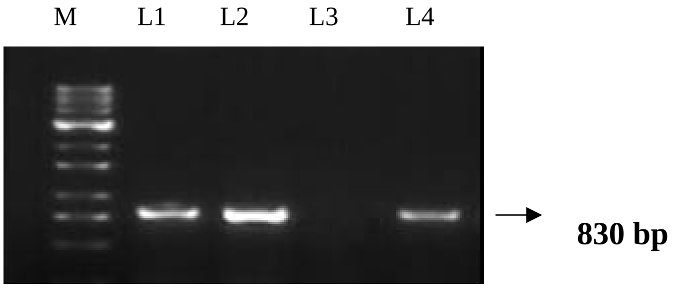

Lane M: 1kb ladder; Lane 1, 2: GBNV inoculated cowpea samples; Lane 3: Negative control; Lane 4: Positive control

Fig S3. Screening the antiviral efficacy of bacterial isolates against GBNV in cowpea (VBN 3)

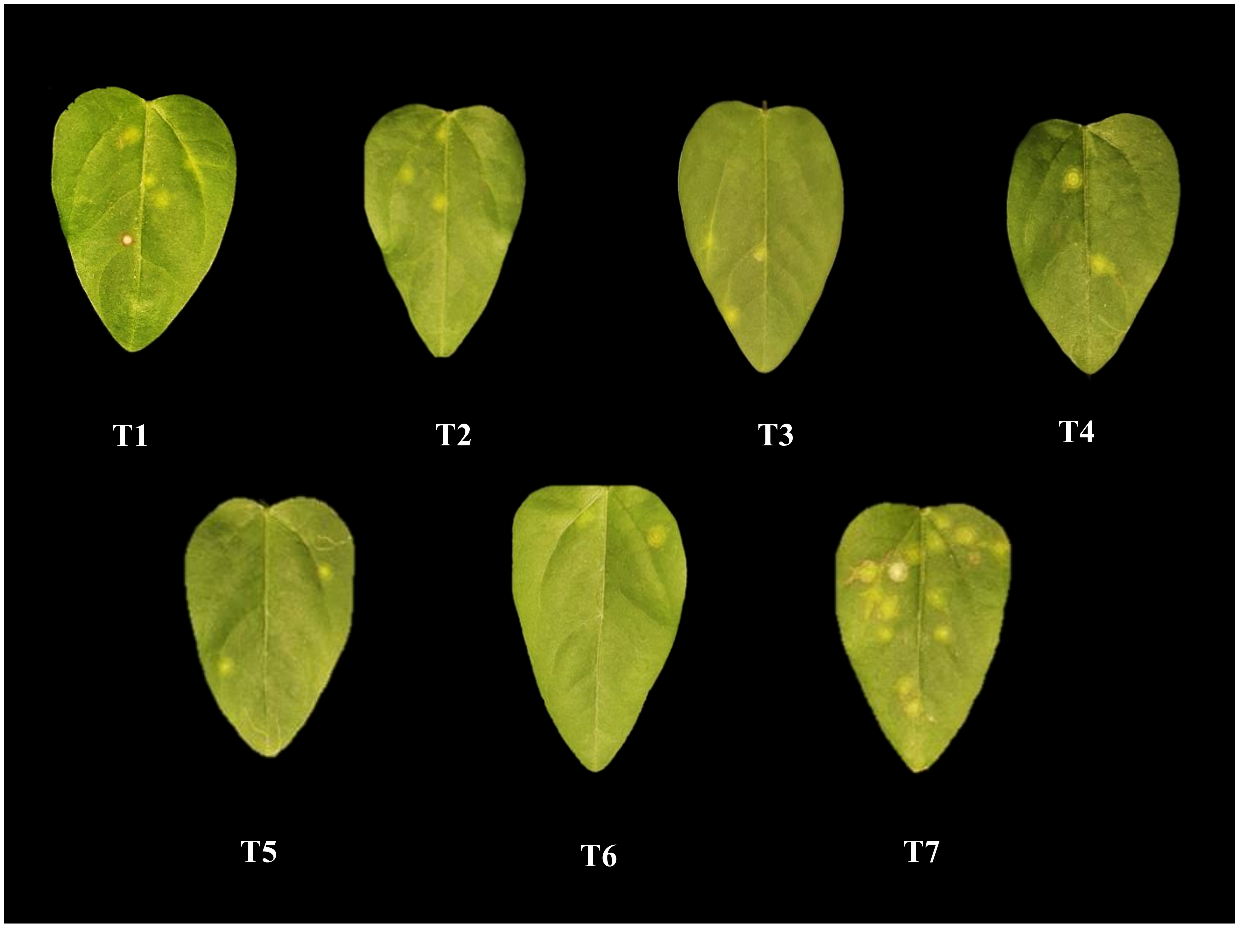

T1- ***Bacillus haynesii*** **IBHB1; T2-** ***Bacillus amyloliquefaciens*** **IBHB2; T3-** ***Stenotrophomonas maltophila*** **IBHB3; T4-*****Bacillus subtilis*** **IBHB4; T5-** ***Pseudomonas aeruginosa*** **IBHB5; T6-** ***Bacilllus subtilis*** **NMB01; T7- Untreated inoculated control**

Fig S4. Root architecture of NMB01 treated vs untreated plants

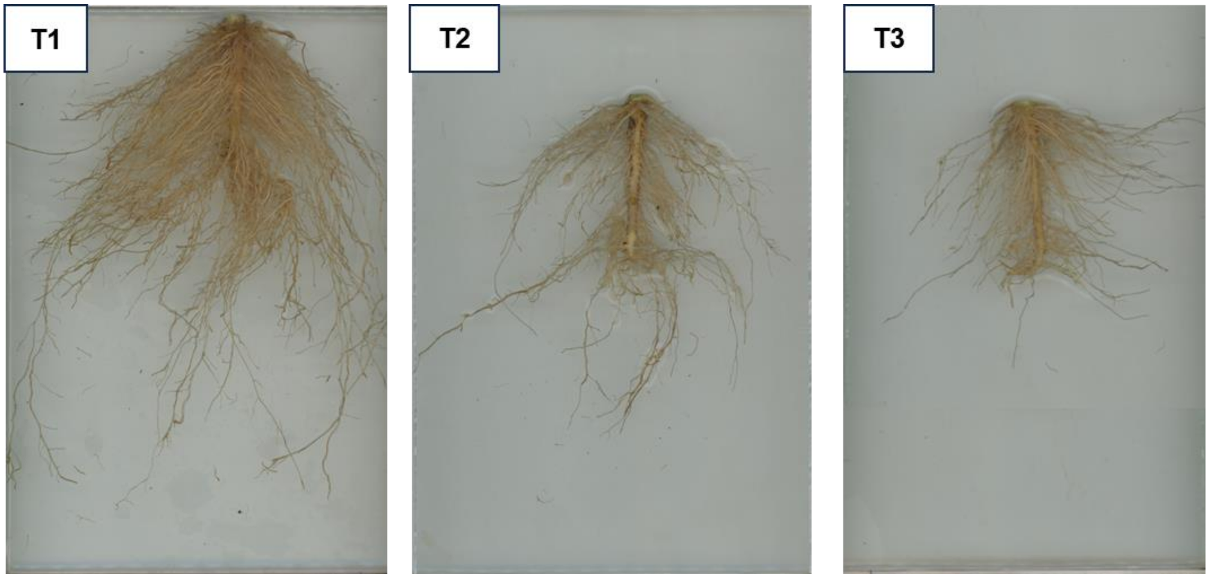

T1- NMB01 + GBNV; T2- Untreated inoculated control, T3- Healthy control

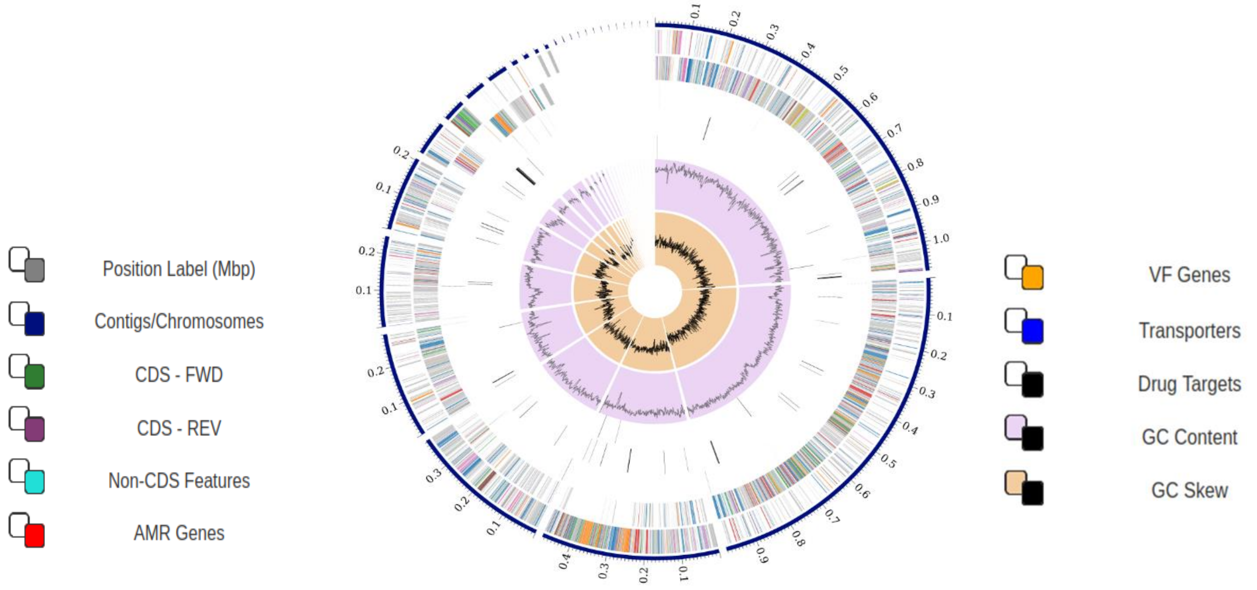

Figure S5. A circular graphical representation showcasing the distribution of genome annotations. The colors of the protein-coding sequences (CDS) on both the forward and reverse strands indicate the specific subsystem to which these genes belong. The circular representation includes various rings, starting from the outermost: contigs, CDS on the forward strand, CDS on the reverse strand, RNA genes, CDS with homology to known antimicrobial resistance genes, CDS with homology to known virulence factors, GC content, and GC skew.

Figure S6a. Subsystem superclass distribution of *B. subtilis* NMB01

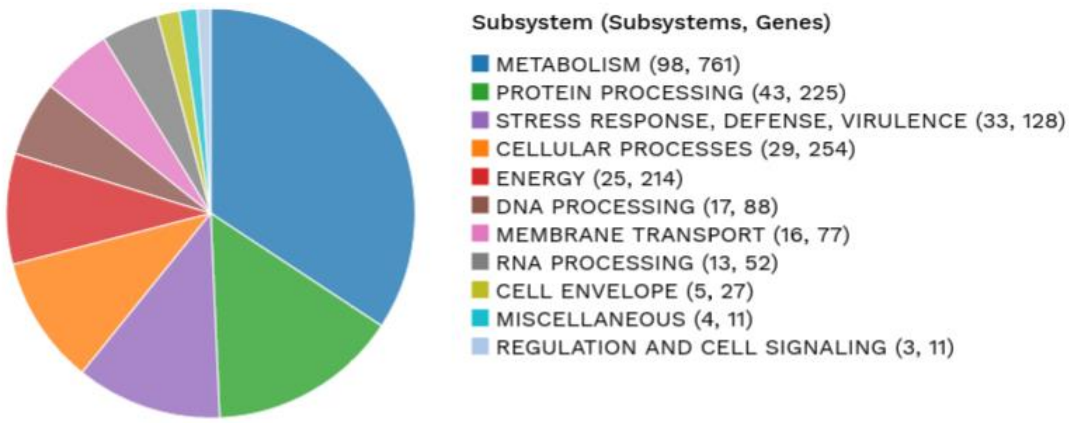

Figure S6b. Gene ontology (GO) annotation and functional classification of *B. subtilis* NMB01 genome.

a. Biological process

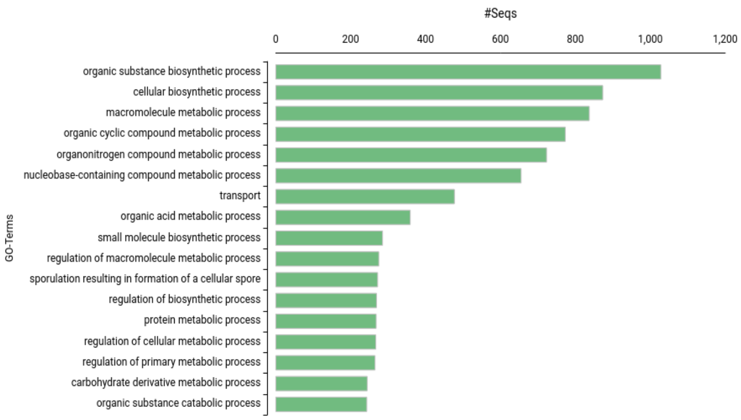

b. Cellular component

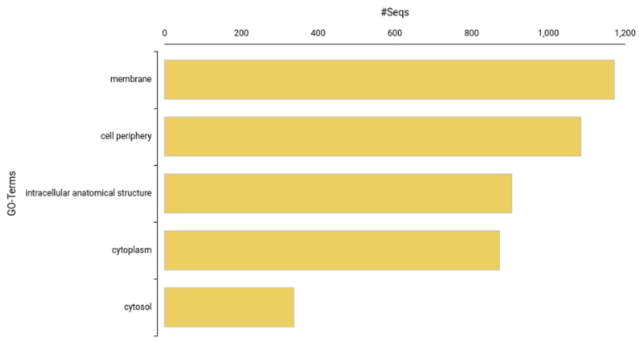

c. Molecular function

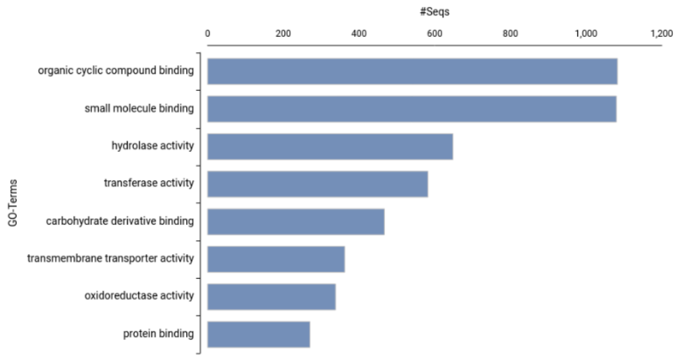

Fig S7a. Pathways of *B. subtilis* genes

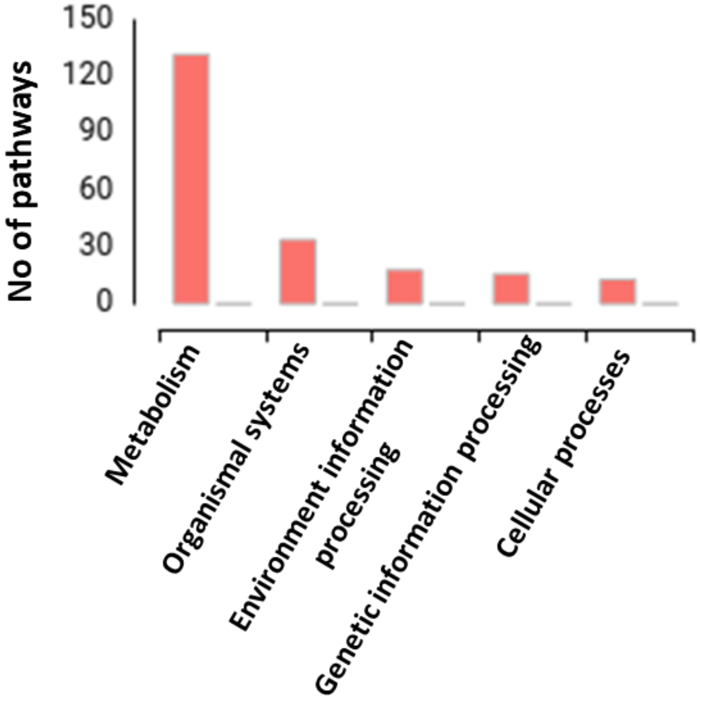

Fig S7b. Pathways Associated with *B. subtilis* Genes

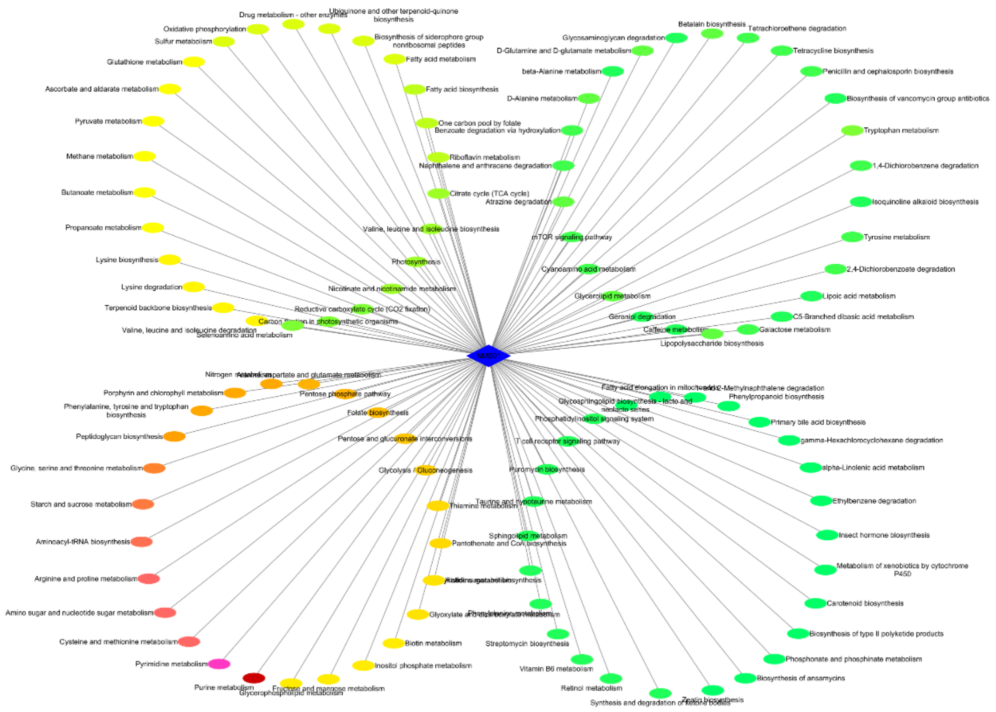

Table. S8. Distribution of core, dispensable and strain specific genes across the strains of *Bacillus subtilis*

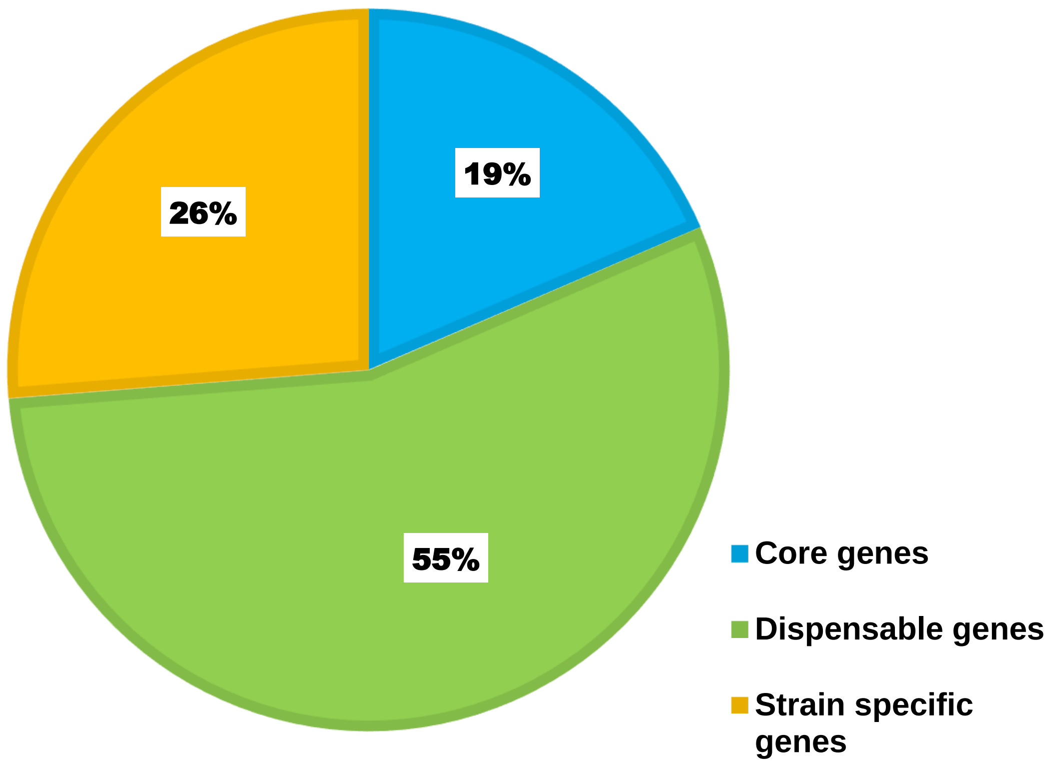

Table S1. The antimicrobial property of the isolates

Isolate	Antimicrobial property	Reference
BYS2	Pathogenic Bacteria	Menjiao et al.,2021
ZD01	Antifungal (*Alternaria solani*)	Zhang et al.,20202
YB04	Antifungal (*Fusarium* Wilt)	Wen xu et al., 2022
XF1	Biological control/ Antimicrobial activity	Gua et al., 2015
UD1022	Antifungal *(Ascochyta medicaginicola*)	Rosier et al., 2023
TR21	Antifungal *(Fusarium oxysporum f. sp.Cubense*)	Yunhao et al.,2022
SG6	Antifungal *(Fusarium graminearum*)	Zhao et al.,2014
RS10	Antibacterial activity (*S. aureus* and *P. syringae*)	Shajid Iqbal et al.,2021
GUCC4	Plant growth promotion and Biocontrol	Wang et al.,2023
MC42	Antiviral (Tobacco Black Shank Disease)	Chunlan shi et al., 2024
PMB102	*Antifungal (Alternaria brassicicola)*	Wu *et al*., 2021
BS16	Antioomycetes (*Aphanomyces cochloides*)	Rajkumar *et al*.,2021
BS16045	Antibacterial and antifungal activity	Jeon *et al*., 2017
BSn5	Antibacterial (*Erwinia carotovora* subsp. *carotovora*)	Yun Deng *et al*., 2011
GQJK2	Antifungal activity	Jinjinma et al.,2017
HD15	Antibacterial activity	Wook Hong et al., 2022
J5	Antifungal activity (*Botrytis cinerea*)	Zhenhua Jia et al., 2017
JCL16	Antifungal activity (*Nocardia seriolae*)	Wang et al., 2022
KC141	Antibacterial activity	Xiaowei et al.,2024

Table S2. List of primers used in the study

S.No	Name of the gene		Primer
1	MAPKK	Forward	5’ AAGCACCAGAAGGACAGAC3’
		Reverse	5’CCTGCGGAAGTGAAGTAAG3’
2	WRKY33	Forward	5’CCACAACAGTCTGAAATGGG3’
		Reverse	5’CAGCAAAGCAATGACTCCAT3’
3	PAL	Forward	5’CAAGGGCTGGTGTGAAAGC3’
		Reverse	5’GTCCTTCCTTGGGCTGCAAC3’
4	NPR1	Forward	5’GACCACGGCATCAAAACTCACC3’
		Reverse	5’GACTTCTTCGCTGATGCTAAGC3’
5	PRI	Forward	5’TCAGGTGGTGTGGCGTTAACTC3’
		Reverse	5’AAGTACCACCACCCGTTGTTGCA3’
6	GBNV CP	Forward	5’GGACCAGATGACTGGACCTTC3’
		Reverse	5’TCGAAAGCTGCAGGGACATT3’
7	Actin	Forward	5’AGGCAGGATTTGCTGGTGATGATGCT3’
		Reverse	5’ATACGCATCCTTCTGTCCCATTCCGA3’

Table S3. Identified secondary metabolite regions using strictness 'relaxed'

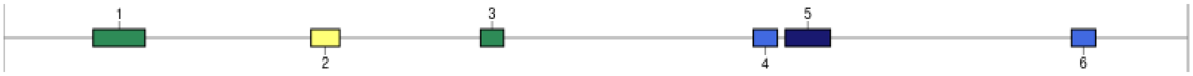

S208	Region	Type	From	To	Most similar known cluster		Similarity (%)
Contig_1	Region 1.1	NRPS	81918	129054	bacillibactin	NRP	100
	Region 1.2	Lanthipeptide-class-i	279951	306176	subtilin	RiPP:Lanthipeptide	100
	Region 1.3	CDPS	434613	455359			
	Region 1.4	Sactipeptide	682885	704496	subtilosin	RiPP:Thiopeptide	100
	Region 1.5	other	711587	753005	bacilysin	Other	100
	Region 1.6	epipeptide	972385	994083	thailanstatin	NRP+Polyketide	10

S208_contig_2

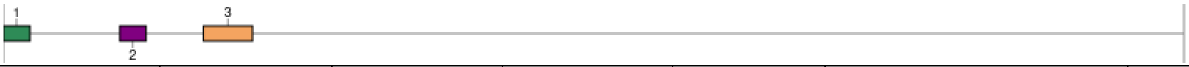

S208	Region	Type	From	To	Most similar known cluster		Similarity (%)
Contig_2	Region 2.1	NRPS	1	22103	plipastatin	NRP	30
	Region 2.2	terpene	96830	118728			
	Region 2.3	T3PKS	166469	207566			

S208_contig_3

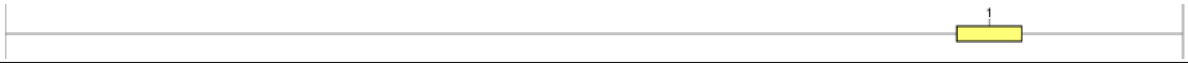

S208	Region	Type	From	To	Most similar known cluster		Similarity (%)
Contig_3	Region 3.1	Lanthipeptide-class-i	384972	411267			

S208_contig_4

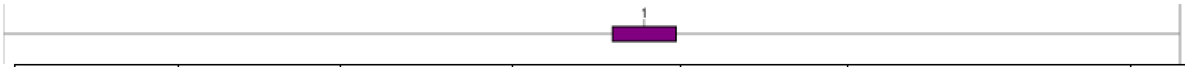

S208	Region	Type	From	To	Most similar known cluster		Similarity (%)
Contig_4	Region 4.1	terpene	198871	219674			

S208_contig_6

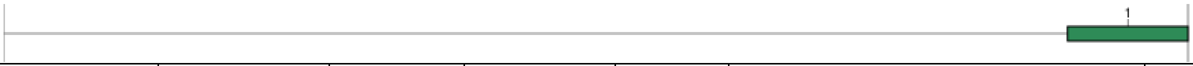

S208	Region	Type	From	To	Most similar known cluster		Similarity (%)
Contig_6	Region 6.1	NRPS	222291	247424	surfactin	NRP:Lipopeptide	39

S208_contig_7

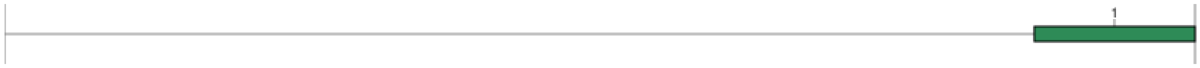

S208	Region	Type	From	To	Most similar known cluster		Similarity (%)
Contig_7	Region 7.1	NRPS	177202	204884	surfactin	NRP:Lipopeptide	43

S208_contig_8

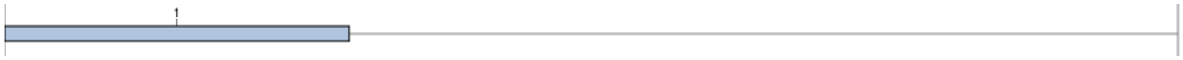

S208	Region	Type	From	To	Most similar known cluster		Similarity (%)
Contig_8	Region 8.1	NRPS,betalactone	1	27547	fengycin	NRP	73

S208_contig_13

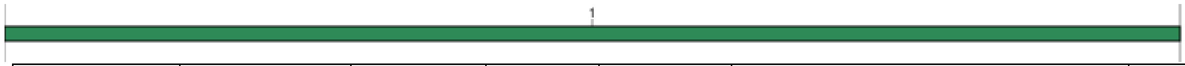

S208	Region	Type	From	To	Most similar known cluster		Similarity (%)
Contig_13	Region 13.1	NRPS	1	13393	plipastatin	NRP	23

S208_contig_14

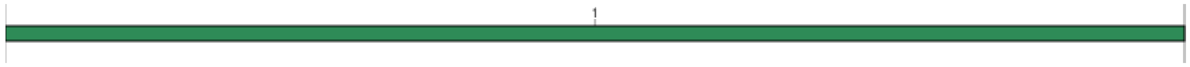

S208	Region	Type	From	To	Most similar known cluster		Similarity (%)
Contig_14	Region 14.1	NRPS	1	9471	plipastatin	NRP	15

S208_contig_15

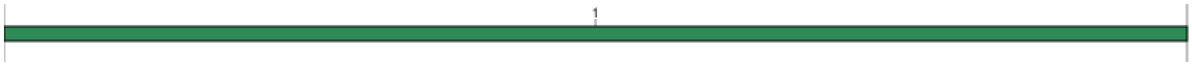

S208	Region	Type	From	To	Most similar known cluster		Similarity (%)
Contig_15	Region 15.1	NRPS	1	8953	surfactin	NRP:Lipopeptide	8

Table S4. Comparison of MAMP genes between *Bacillus subtilis* isolates

Isolate	Peptidoglycan	Flagellin	Elongation factor
BYS2	7	2	6
ZD01	11	2	6
YB04	16	2	6
XF1	7	2	6
UD1022	4	2	8
TR21	22	2	6
SG6	12	2	7
RS10	15	3	8
GUCC4	13	2	6
MC42	14	2	6
PMB102	20	2	6
BS16	7	1	9
BS16045	8	3	5
BSn5	8	1	7
GQJK2	6	2	7
HD15	17	2	6
J5	8	1	8
JCL16	19	2	6
KC141	15	2	6
NMB01	9	3	6

Table S5. Comparison of hydrolytic genes between *Bacillus subtilis* isolates

Name of the isolate	Glucanase	Xylanase	Arabinase	Amylase	Glucosidase	Chitosanase	Tpx	Phosphate ABC	Oligopeptide ABC	Acetolactate	Acetoin dehydrogenase
**BYS2**	2	2	0	2	8	1	1	5	7	4	3
**ZD01**	2	3	0	2	8	1	2	6	7	3	3
**YB04**	2	2	0	2	5	1	2	5	9	4	3
**XF1**	3	2	1	1	3	2	0	6	6	4	2
**UD1022**	2	2	0	1	4	1	0	7	0	4	0
**TR21**	2	2	0	2	4	1	2	6	9	4	3
**SG6**	2	2	0	2	9	1	1	0	0	4	1
**RS10**	2	2	0	3	9	1	2	6	8	4	1
**GUCC4**	2	2	0	2	9	1	2	7	8	5	3
**MC42**	2	2	0	2	9	1	2	6	0	4	3
**PMB102**	2	2	0	2	8	1	2	6	7	4	3
**BS16**	2	2	0	1	7	1	0	7	0	4	0
**BS16045**	1	2	0	1	9	1	0	0	0	4	1
**BSn5**	7	3	1	1	15	1	0	6	6	4	2
**GQJK2**	2	3	0	1	9	1	0	6	6	4	0
**HD15**	2	2	0	2	8	1	2	7	7	4	3
**J5**	2	2	0	1	7	1	0	7	7	4	0
**JCL16**	2	2	0	2	11	1	2	7	7	4	3
**KC141**	2	2	0	2	9	1	2	6	6	4	3
**NMB01**	**2**	**2**	**15**	**2**	**9**	**1**	**2**	**5**	**11**	**3**	**5**
